# The Arabidopsis DREB2 genetic pathway is constitutively repressed by basal phosphoinositide-dependent phospholipase C coupled to diacylglycerol kinase

**DOI:** 10.3389/fpls.2013.00307

**Published:** 2013-08-08

**Authors:** Nabila Djafi, Chantal Vergnolle, Catherine Cantrel, Wojciech Wietrzyñski, Elise Delage, Françoise Cochet, Juliette Puyaubert, Ludivine Soubigou-Taconnat, Delphine Gey, Sylvie Collin, Sandrine Balzergue, Alain Zachowski, Eric Ruelland

**Affiliations:** ^1^Physiologie Cellulaire et Moléculaire des Plantes, CNRS EAC7180Paris, France; ^2^Physiologie Cellulaire et Moléculaire des Plantes, UPMC-Univ Paris06 UR5Paris, France; ^3^Unité de Recherche en Biologie Végétale, UMR INRA 1165, Université d'Evry Val d'Essonne, ERL CNRS 8196Evry Cedex, France

**Keywords:** phospholipase C, diacylglycerol kinase, phosphatidic acid, phospholipase D, DREB2 transcription factors, abiotic stress, lipid signaling

## Abstract

Phosphoinositide-dependent phospholipases C (PI-PLCs) are activated in response to various stimuli. They utilize substrates provided by type III-Phosphatidylinositol-4 kinases (PI4KIII) to produce inositol triphosphate and diacylglycerol (DAG) that is phosphorylated into phosphatidic acid (PA) by DAG-kinases (DGKs). The roles of PI4KIIIs, PI-PLCs, and DGKs in basal signaling are poorly understood. We investigated the control of gene expression by basal PI-PLC pathway in *Arabidopsis thaliana* suspension cells. A transcriptome-wide analysis allowed the identification of genes whose expression was altered by edelfosine, 30 μM wortmannin, or R59022, inhibitors of PI-PLCs, PI4KIIIs, and DGKs, respectively. We found that a gene responsive to one of these molecules is more likely to be similarly regulated by the other two inhibitors. The common action of these agents is to inhibit PA formation, showing that basal PI-PLCs act, in part, on gene expression through their coupling to DGKs. Amongst the genes up-regulated in presence of the inhibitors, were some *DREB2* genes, in suspension cells and in seedlings. The *DREB2* genes encode transcription factors with major roles in responses to environmental stresses, including dehydration. They bind to C-repeat motifs, known as Drought-Responsive Elements that are indeed enriched in the promoters of genes up-regulated by PI-PLC pathway inhibitors. PA can also be produced by phospholipases D (PLDs). We show that the *DREB2* genes that are up-regulated by PI-PLC inhibitors are positively or negatively regulated, or indifferent, to PLD basal activity. Our data show that the DREB2 genetic pathway is constitutively repressed in resting conditions and that DGK coupled to PI-PLC is active in this process, in suspension cells and seedlings. We discuss how this basal negative regulation of *DREB2* genes is compatible with their stress-triggered positive regulation.

## Introduction

Besides their structural role as membrane constituents, lipids are signal mediators that recruit target enzymes to a specific membrane and this can lead to the functional activation or inhibition of the recruited proteins (Wang et al., 2006). Phosphoglycerolipids are a major class of signaling lipids, their mediating action resulting either from the action of lipid kinases or from the action of phospholipases (Janda et al., 2013). Phosphatidylinositol-4-kinases (PI4Ks) phosphorylate phosphatidylinositol into phosphatidylinositol-4-P (PI4P) that can be further phosphorylated into phosphatidylinositol-4,5-bisP (PI(4,5)P_2_) by PI4P-5-kinases. The PI4P and PI(4,5)P_2_ phosphoinositides can be bound by proteins, more particularly through the pleckstrin homology (PH) domain. For instance, the PH domain of EDR2 (Enhanced disease resistance 2) protein that regulates plant defense and cell death, binds PI4P *in vitro* (Vorwerk et al., 2007). PI(4,5)P_2_ is also known to regulate actin cytoskeleton and vesicle trafficking, and this might be important for polarized growth of root hair and pollen tube (Monteiro et al., 2005; Stenzel et al., 2008; Thole and Nielsen, 2008; Zhao et al., 2010). PI(4,5)P_2_ also binds some phospholipases D (PLDs) and has a positive impact on their activities as a cofactor (Qin and Wang, 2002). PLDs hydrolyze structural lipids, such as phosphatidylcholine (PC) and phosphatidylethanolamine (PE), into phosphatidic acid (PA). PI(4,5)P_2_ is also substrate of phosphoinositide-dependent PLCs (PI-PLCs) that will hydrolyze it into diacylglycerol (DAG) and inositol triphosphate (InsP_3_). DAG can be phosphorylated into PA by diacylglycerol kinases (DGKs) and soluble InsP_3_ can be further phosphorylated into highly phosphorylated inositol (Stevenson-Paulik and Phillippy, 2010). The relative importance of InsP_3_ (or its derivatives), of DAG and of PA in PI-PLC dependent responses is poorly understood. DGKs have been shown to be activated in response to host–pathogen interactions, to elicitors such as xylanase, but also in response to abiotic stresses such as salinity, osmotic stress, and cold (Ruelland et al., 2002; Arisz et al., 2009). The coupling of PI-PLC and DGK activities has been shown to occur in response to cold or chitosan elicitor (Bargmann and Munnik, 2006). However, whether DGKs act to attenuate a messenger (DAG) or to produce one (PA) in the PI-PLC module is not established. Since genes encoding proteins homologous to PKC, the archetypal mammal DAG-binding protein, have not been found in plant genomes, it is assumed that the active lipid messenger produced by PI-PLC pathway is PA, through the coupling with DGK. Indeed PA target proteins have been identified (Wang et al., 2006). Yet this does not mean that DAG has no role. The C1 domain is responsible for DAG binding in mammalian PKC. C1-domain bearing proteins exist in plants (Janda et al., 2013). More data are thus necessary to document the role of PA produced by DGKs in the PI-PLC module.

Besides a role in response to an elicitation, lipid signaling could also occur in non-stimulated cells, thus participating in basal signaling (Boss et al., 2010). A so-called non-stimulated cell is not a cell in which no intracellular signaling occurs. On the contrary, a non-stimulated cell is a cell where its steady-state is attained through the action of basal signals, some of which participate actively and constitutively in repressing or stimulating downstream events, in particular, gene expression. Therefore, we investigated the involvement of lipid signaling, especially that of the PI-PLC pathway, in the basal regulation of gene expression. In Arabidopsis, PI-PLCs are encoded by a family of 9 members (Pokotylo et al., 2014) and redundancy of PI-PLC proteins has been suggested. In a single tissue, where several isoforms are expressed, they all appear to be functionally identical (Hunt et al., 2004) and phenotypes of single mutants are scarce. When studying basal signaling, the use of mutants is not necessarily appropriate since plants can constitutively activate compensatory mechanisms. Thus, a pharmacological approach where all the isoforms are inhibited at the same time, has been proposed (Powis et al., 1992; Horowitz et al., 2005; Wong et al., 2007; Kelm et al., 2010) and was utilized in the current study. In this way, genes were identified whose basal expression was altered in the presence of PI-PLC inhibitors, namely edelfosine or U73122. In experiments in presence of wortmannin, an inhibitor of type III-phosphatidylinositol 4-kinases (PI4KIIIs) that provide substrates to PI-PLC, or R59022, a DGK inhibitor that prevents PA synthesis from DAG, we found a strong statistical bias in favor of genes that were similarly regulated by edelfosine and these inhibitors. These genes are likely to be regulated by the basal PA produced by a PI-PLC pathway. Interestingly, some *DREB2* genes are amongst the genes up-regulated when basal PI-PLC is inhibited. Their target genes are also over-represented in the genes altered by inhibitor treatment. Our data establish that the DREB2 genetic pathway is constitutively repressed in Arabidopis suspension cells and seedlings, and that DGK coupled to PI-PLC is active in this process. Interestingly, the *DREB2* genes up-regulated by PI-PLC inhibitors are not necessarily up-regulated when basal PLD-produced PA is lowered, suggesting there are no general “PA-responsive genes” but genes responsive to DGK-produced PA and genes responsive to PLD-produced PA. This article is one of the first reports revealing a role for plant DGKs. Because of the major role of DREB2 transcription factors in plant responses to dehydration, this role appears of high importance.

## Results

### Identification of edelfosine-controlled genes

**Figure 1:**
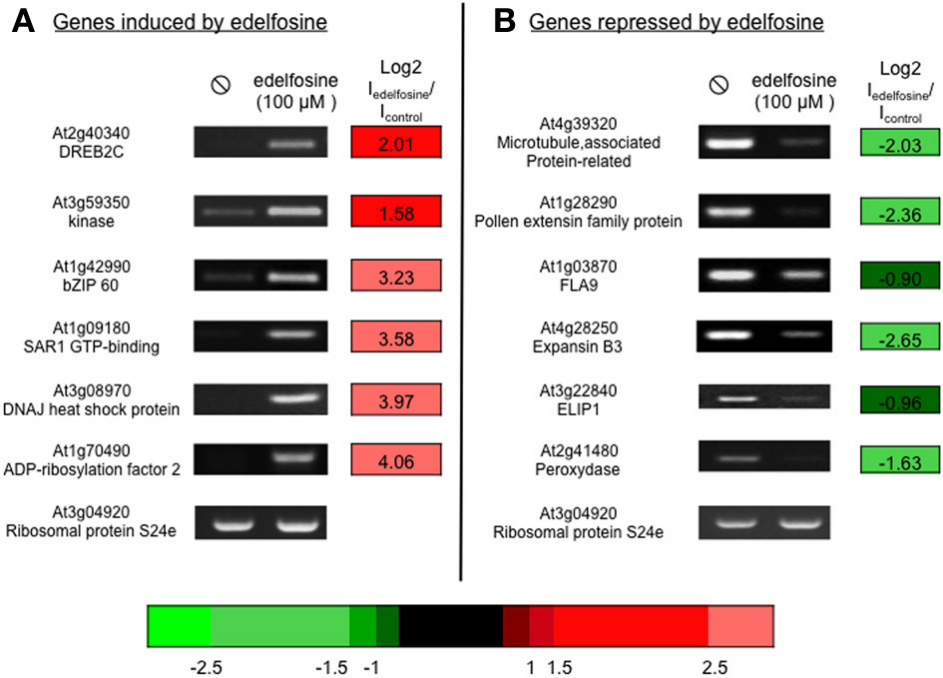
**Edelfosine alters basal gene expression**. We verified on a set of genes that edelfosine could stimulate **(A)** or repress **(B)** basal expression. Arabidopsis suspension cells were treated with 100 μM edelfosine for 4 h, at 22°C, before harvesting. Transcript levels were estimated by reverse transcriptase-PCR, using an appropriate number of cycles. The microarray data of the corresponding genes are expressed as log2 (I_edelfosine_/I_control_). The color scale for microarray data is indicated.

Arabidopsis suspension cells were incubated with 100 μM edelfosine and harvested after 4 h. The transcriptome of edelfosine-treated cells was compared to that of non-treated cells using CATMA chips (Hilson et al., 2004). Of the 26139 CATMA probes leading to a signal, 412 were induced by edelfosine while 515 were repressed (listed in Supplemental Table S1A). The expression of a selection of genes, either induced or repressed by edelfosine, was verified by RT-PCR (Figure 1) thus confirming their response to the inhibitor.

A transcriptomic study of cells treated with U73122, another widely used PI-PLC inhibitor (Horowitz et al., 2005) was performed. As a control, U73343, an inactive analog of U73122, was used. Of the 38383 CATMA probes leading to a signal, 745 were repressed by U73122 and 1091 were induced by U73122 (listed in Supplemental Table S1B).

Microarray data obtained from edelfosine-treated cells were then crossed with those obtained from cells treated with U73122. Twenty-one thousand three hundred and sixty nine probes were identified which had a signal in both experiments. A probe can be up-regulated, down-regulated, or not regulated by edelfosine or by U73122 (when compared to U73343). Therefore, combining the possible responses leads to 9 categories of behavior for each probe. A contingency table shows the number of probes associated with each category (Table 1). The experimental numbers can be compared to the theoretical numbers assuming that sensitivities to edelfosine and to U73122 are independent and the ratio between observed and theoretical values was calculated. A Pearson's Chi-squared test clearly showed (*p*-value less than 2.2e-16) that the sensitivity to edelfosine was not independent from the sensitivity to U73122. We identified 127 genes induced by edelfosine and U73122, and 122 genes repressed by both molecules (listed in Supplemental Table S1C); this represents 10- and 17-fold more probes than expected theoretically in case of independent actions, respectively. On the contrary, the genes regulated in different ways by each inhibitor were very much under-represented: for instance, there was no gene repressed by U73122 and induced by edelfosine. This clearly shows the effects of edelfosine and of U73122 are not independent, and most likely represent the consequence of the common action of these molecules, i.e., the inhibition of PI-PLC. A Venn diagram representing the overlaps between gene regulated by U73122 or edelfosine is in Supplemental Figure S1A.

**Table 1:** **Contingency table of the expression of genes in response to edelfosine or U73122 treatments (Bonferroni correction *p*-value < 0.05)**.

**Observed (Theoretical) ratio**	**U73122 > U73343**	**U73122 <> U73343**	**U73122 < U73343**	**Total**
Edelfosine > control	127	240	0	376
	(12.93)	(355)	(10.33)	
	**9.90**	**0.54**	**0**	
Edelfosine <> control	600	19466	462	20528
	(702)	(19265)	(561)	
	**0.85**	**1.02**	**0.82**	
Edelfosine < control	3	340	122	465
	(15.9)	(436.4)	(12.71)	
	**0.19**	**0.51**	**16.53**	
Total	731	20056	584	21369

*For each category, the number of observed genes can be compared to that of theoretical genes, indicated between brackets, considering that the expression of genes in response to each of the 2 molecules is independent. The ratio “number of observed genes” vs. “theoretical number” is in bold*.

### Edelfosine-controlled genes are also controlled by 30μM wortmannin, a type III-Pi4K inhibitor

We recently showed that PI-PLC substrates are provided by type III-PI4Ks (Delage et al., 2012). These enzymes that catalyze the formation of PI4P, are inhibited by micromolar concentrations of wortmannin (Krinke et al., 2007). PI-PLCs can act either by the molecules they produce, but also by the fact they consume substrates and lower their concentrations (See working model in Supplemental Figures S2A,B). If the effect of PI-PLCs is to deplete phosphoinositides, then edelfosine (that—by inhibiting PI-PLCs—would increase phosphoinositide level) and 30 μM wortmannin (that inhibits phosphoinositide production) should have reverse effects on gene expression (Supplemental Figures S2B–D). If the action on gene expression is through PA, then edelfosine (inhibiting phospholipase activity) and wortmannin (inhibiting phospholipase activity by preventing substrate synthesis) would have similar effects. We had previously performed a microarray experiment using Arabidopsis suspension cells treated by 30 μM wortmannin (Krinke et al., 2007). Because wortmannin can also inhibit phosphatidylinositol-3-kinases (PI3Ks), but at a lower (nanomolar) concentration, comparing the effects of 30 μM wortmannin (W30) to those of 1 μM wortmannin (W1) identifies genes for which the effect of W30 is not attributable to an inhibition of PI3Ks.

**Figure 2:**
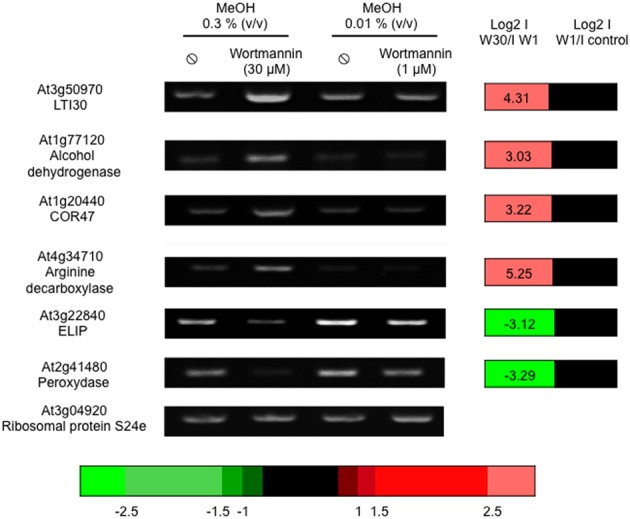
**Wortmannin (30 μM) alters basal gene expression**. Cells were treated with 1 or 30 μM wortmannin for 4 h, at 22°C, before harvesting. Transcript levels were estimated by reverse transcriptase-PCR, using an appropriate number of cycles. The microarray data of the corresponding genes are expressed as log2 (I_W30_/I_W1_) and log2 (I_W1_/I_control_). The color scale for microarray data is indicated.

Of the 25242 microarray probes with a signal, 2942 genes Supplemental Table S1D) showed a differential expression in the presence of W30 *vs*. W1: 1629 were more induced in the presence of W30 compared to W1 while 1313 genes had the opposite regulation. We verified, in an independent experiment that the expression of several genes was indeed altered by W30 (Figure 2). We then crossed the microarray data obtained from edelfosine-treated cells with those obtained from cells treated with W30. We identified 24539 probes with a signal in both experiments. As above, a contingency table was filled with the number of probes observed for each category (Table 2) and these numbers were compared to the theoretical ones assuming sensitivities to edelfosine and to W30 are independent. A Pearson's Chi-squared test clearly showed (*p*-value less than 2.2e-16) that the sensitivity to edelfosine was not independent from the sensitivity to W30. There were 10-fold more probes that were either up-regulated (229) or down-regulated (367) by edelfosine and by W30 than theoretically expected. On the contrary, there was an under representation of genes for which edelfosine and W30 have opposite effects. A Venn diagram representing the overlaps between gene regulated by edelfosine or W30 is in Supplemental Figure S1B. These results confirmed that there was a functional coupling between type-III-PI4K and PI-PLC leading to the control of gene expression, and that basal PI-PLC acts on gene expression via its products and not by depleting phosphoinositides. The same analysis was done using the transcriptome of U73122-dependent genes, and the conclusion reached was the same (Supplemental Table S2A).

**Table 2:** **Contingency table of the expression of genes sensitive in response to edelfosine or 30 μM wortmannin treatments**.

**Observed (Theoretical) ratio**	**Edelfosine > control**	**Edelfosine <> control**	**Edelfosine < control**	**Total**
W30 > W1	229	1059	12	1300
	(21.83)	(1251.05)	(27.12)	
	**10.5**	**0.88**	**0.44**	
W30 <> W1	173	21307	133	21613
	(362.87)	(20799.18)	(450.95)	
	**0.48**	**1.02**	**0.29**	
W30 < W1	10	1249	367	1626
	(27.30)	(1564.77)	(33.93)	
	**0.37**	**0.8**	**10.81**	
Total	412	23615	512	24539

*For each category, the number of observed genes can be compared to that of theoretical genes, indicated between brackets, considering that the expression of genes to each of the 2 molecules is independent. The ratio “number of observed genes” vs. “theoretical number” is in bold*.

The pool of genes regulated the same way by edelfosine and W30 (Supplemental Table S1E), thus controlled by PI-PLC through its products, were classified according to their associated biological processes (Provart and Zhu, 2003). When compared to the Arabidopsis whole genome set, there was a significant over representation of “responses to abiotic and biotic stimulus” and “response to stress.” These categories were also over represented in the edelfosine—and W30—repressed genes, for which the most over represented category was “electron transport or energy pathways” (Supplemental Figure S3).

An *in silico* promoter analysis was carried out to find *cis*-elements that were over represented in the promoters of these genes (Table 3). In the group of genes induced by both chemical agents, *cis*-elements such as C-repeat/Drought Responsive Elements (CRT/DRE), G box, and Coupling element 3 motifs were present. These elements are typical of stress responsive genes and have been involved—*inter allia*—in responses to drought, cold, and abscisic acid (ABA; Williams et al., 1992; Shinozaki and Yamaguchi-Shinozaki, 2000; Gómez-Porras et al., 2007).

**Table 3:** **Motifs over represented in the genes induced or repressed by edelfosine and 30 μM wortmannin**.

**Motif**	**Closest motif in database**		
	**Name**	**Sequence**	**References**
**EDELFOSINE- AND W30-INDUCED**			
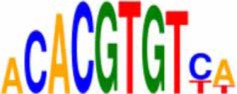	ACGT element, G - box	CACGTG	Williams et al., 1992
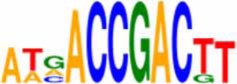	CRT/DRE-like motif	DRCCGACNW	Shinozaki and Yamaguchi-Shinozaki, 2000
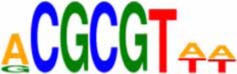	Coupling element 3 - like	ACGCGTGTCCTC	Gómez-Porras et al., 2007
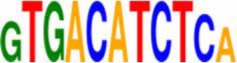	TCA1MOTIF – like	TCATCTTCTT	Goldsbrough et al., 1993
**EDELFOSINE- AND W30-REPRESSED**			
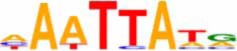	ATHB6COREAT	CAATTATTA	Himmelbach et al., 2002
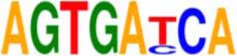	PIATGAPB	GTGATCAC	Chan et al., 2001
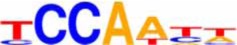	Not described in database	

*Promoter sequences (−1000 bp) were retrieved from TAIR and scanned for 4 to 10 b pb motifs. For each motif, its occurrence in the groups of interest was compared to that in the promoters of the whole genome. The table shows only significantly over represented motifs (p-value < 10^−5^; Chi-squared test)*.

### Edelfosine-controlled genes are also controlled by R59022, a DGK inhibitor

Arabidopsis cells were also treated by R59022, a DGK inhibitor (Ruelland et al., 2002; Laxalt et al., 2007). We identified 205 probes as R59022-repressed and 294 as R59022-induced (Supplemental Table S1F). We crossed these data with those of the edelfosine experiment, and identified 25662 probes with a signal in both experiments. A Pearson's Chi-squared test (*p*-value < 2.2e-16; chi-squared 5684) indicated that R59022 sensitivity was not independent of edelfosine sensitivity. There were indeed 24-fold more probes up-regulated or down-regulated by edelfosine and by R59022 than expected if the sensitivities to the two chemicals were independent (Supplemental Table S2B). Supplemental Table S1G lists these genes. A Venn diagram representing the overlaps between genes regulated by R59022 or edelfosine is in Supplemental Figure S1C.

**Figure 3:**
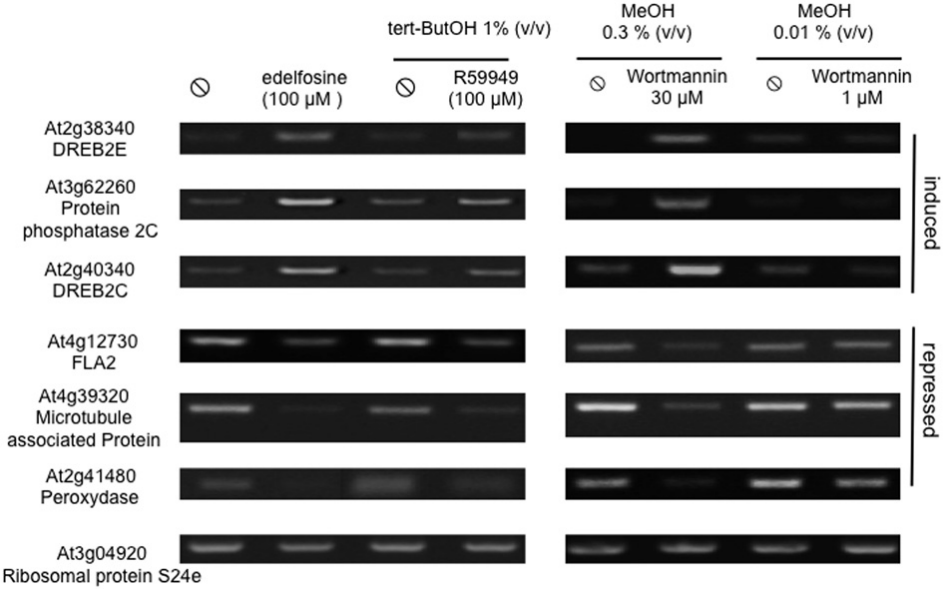
**Edelfosine, W30, and R59949 can have similar effects on basal gene expression**. We verified on a set of genes that edelfosine, wortmannin, and R59949 could, separately, stimulate or repress basal expression. Cells were treated with edelfosine, wortmannin, or R59949 for 4 h, at 22°C, before harvesting. Transcript levels were estimated by reverse transcriptase-PCR, using an appropriate number of cycles.

Finally, genes that were regulated the same way by W30, edelfosine, and R59022 were identified. Fifty-three genes were induced by the three inhibitors, a number much higher than expected (2) if the effects of these molecules were independent (Supplemental Table S2C). Similarly, 47 genes were repressed by each molecule. This is 29-fold more than expected. The full list of genes regulated the same way by W30, edelfosine, and R59022 is given in Supplemental Table S1H. We verified on some of these genes that they were indeed sensitive to edelfosine, to W30 and also to R59949, another DGK inhibitor (Jiang et al., 2000) (Figure 3). A Venn diagram representing the overlaps between genes induced by R59022, or edelfosine, or W30 is in Supplemental Figure S1D, while a diagram representing the overlaps between genes repressed by R59022, or edelfosine, or W30 is in Supplemental Figure S1E.

All inhibitors used in this work prevent PA formation by the PLC pathway, by directly acting either on DGK (R59022/R59949) or on PI-PLC (edelfosine/U73122) or by acting on the substrate provided to PI-PLC (W30). The genes regulated similarly by the three treatments can be considered as regulated by PA produced by the PLC module.

### The DREB2 genetic pathway is constitutively inhibited by basal PI-PLC in suspension cells and seedlings

**Figure 4:**
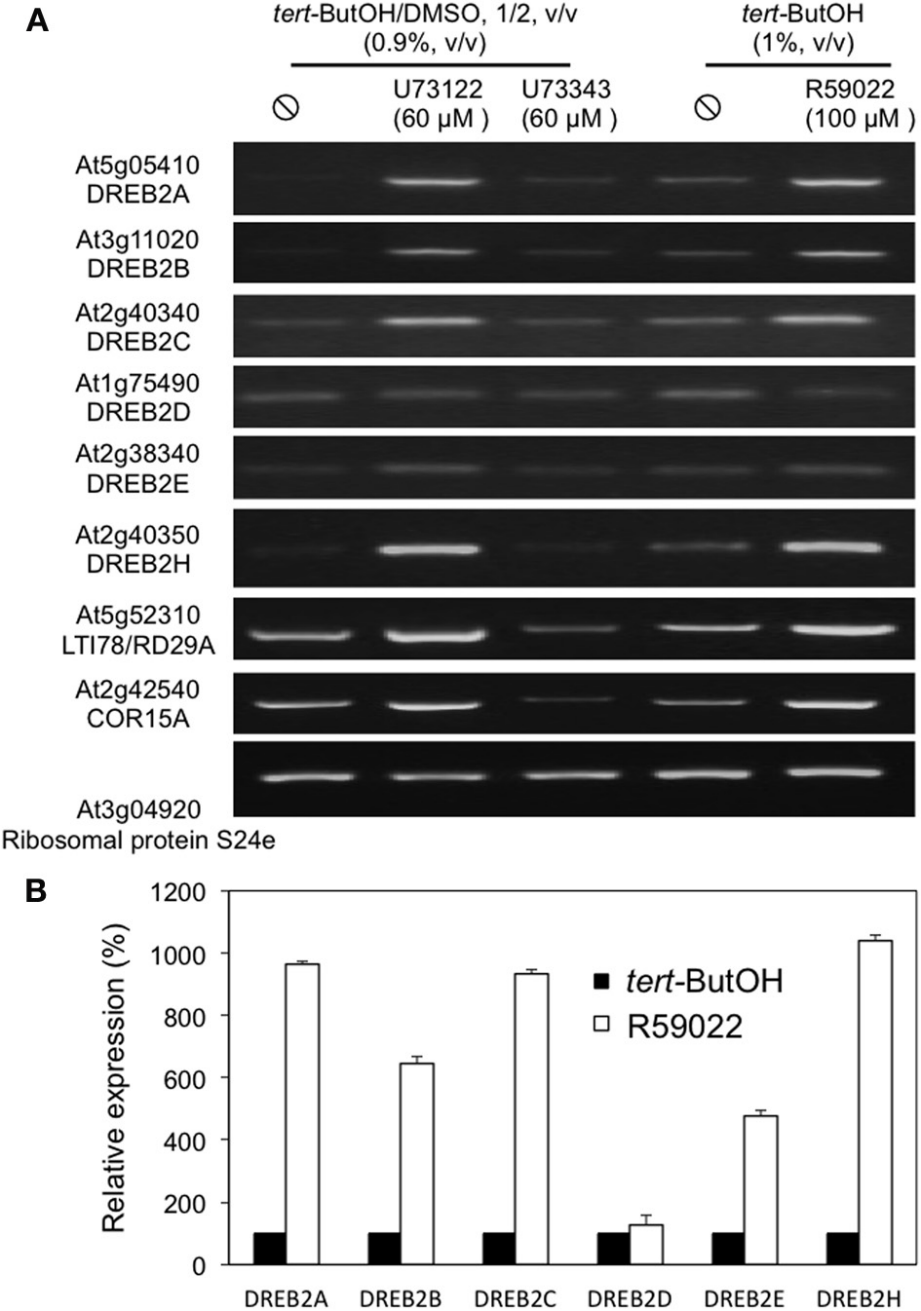
**Effects of inhibitors of PI-PLC or DGK on the expression levels of *DREB2* genes and two DREB-target genes**. **(A)** Transcript levels were estimated by reverse transcriptase-PCR, using an appropriate number of cycles. **(B)** Transcript level of DREB2 genes measured by qPCR, and expressed as % of the level in solvent treated cells. Cells were treated with inhibitors for 4 h, at 22°C, before harvesting.

In Figure 3, two genes encoding DREB2 proteins, which are transcription factors that bind the CRT/DRE motifs, were present as being induced by the inhibitors. We therefore analysed the response of all *DREB2* genes to inhibitor treatments by RT-PCR (Figure 4A). We were not able to amplify *DREB2F* nor *DREB2G* cDNAs. All other *DREB2* genes, but not *DREB2D*, showed an induction in presence of U73122 or R59022. Quantitative real-time PCR (qPCR) confirmed R59022-induced *DREB2A* expression (Figure 4B).

**Figure 5:**
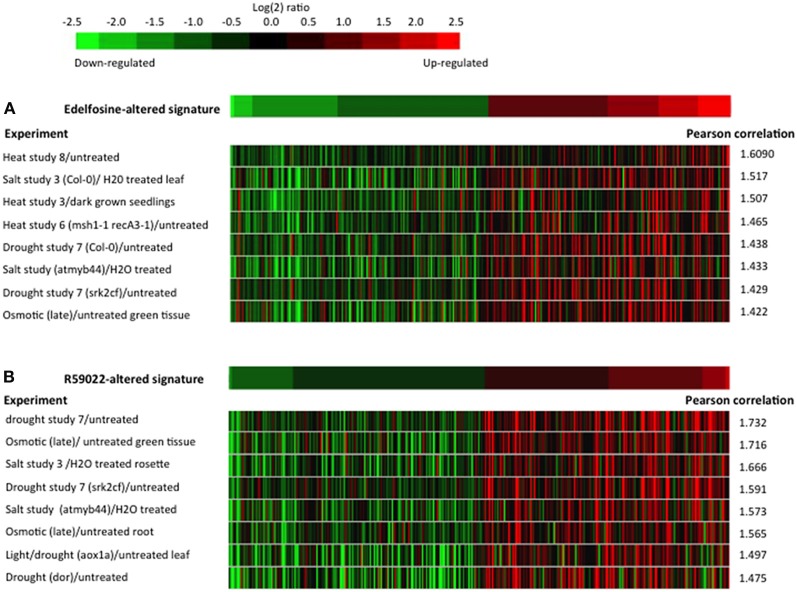
**Similarity between the edelfosine-responsive or R59022-responsive transcriptomes and the stress-responsive transcriptomes**. **(A)** The 200 genes that are most up-regulated by edelfosine and the 200 genes that are most down-regulated by edelfosine were used as a signature to search for the transcriptome experiments with the highest similarity. **(B)** The 200 genes that are most up-regulated by R59022 and the 200 genes that are most down-regulated by R59022 were used as a signature to search for the transcriptome experiments with the highest similarity. The similarity search was performed against the 789 experiments classified as “stress” by Genevestigator (Hruz et al., 2008). The experiments are sorted according to the Pearson's correlation. The expressions of the signature genes in the 8 most similar experiments are shown in color-scale. *msh1-1recA3-1*, *atmyb44*, *srk2cf*, *aox1*, *dor* are mutant plants in *MSH1* and *RecA3*, *MYB transcription factor 44* (Jung et al., 2008), *SnRK2C* and *SnRK2F* (Mizoguchi et al., 2010), *alternative oxidase1* (Giraud et al., 2008), and the *F-box protein DOR* (Zhang et al., 2008), respectively.

Lists of edelfosine- and R59022-altered genes were used as signatures to interrogate the microarray experiments using the Genevestigator similarity search program (Hruz et al., 2008). In both instances, of the 789 experiments classified as “stress” by Genevestigator, the eight top experiments with highest similarity are experiments in which plants were treated either by salt, drought, or heat, in wild-type plants or in mutant genetic backgrounds (Figure 5). These are the very stresses in which DREB2s is reported to act as main transcription factors (Sakuma et al., 2006; Mizoi et al., 2013). As to DREB2 target genes, Mizoi et al. (2013) published a list of genes whose expression is altered in Arabidopsis when Arabidopsis or Soybean DREB2A, in their native or constitutively active forms, are over expressed. These genes are considered as being downstream DREB2A, and to be DREB2 direct or indirect target genes. We compared our list of edelfosine-altered genes with that of these genes. Among 515 edelfosine-repressed genes, 281 genes are altered in at least one of the DREB2A over expressing mutants, and among 412 edelfosine-induced genes, 211 are altered in at least one of the over expressing mutants (Supplemental Table S3A). More than half of edelfosine responsive genes are downstream of DREB2A. Similarly, of 205 R59022-repressed genes and 294 R59022-induced genes, 119 and 149, respectively, are downstream DREB2A (Supplemental Table S3B).

The CRT/DRE motif was first identified by deleting the promoters of *LTI78*/*RD29A* (Yamaguchi-Shinozaki and Shinozaki, 1994) and *COR15A* (Baker et al., 1994). This makes those 2 genes to be considered as the canonical DREB2 target genes. We analysed their responses to inhibitors of the PI-PLC pathway, and found that inhibiting PI-PLC or DGK led to their expression (Figure 4A).

**Figure 6:**
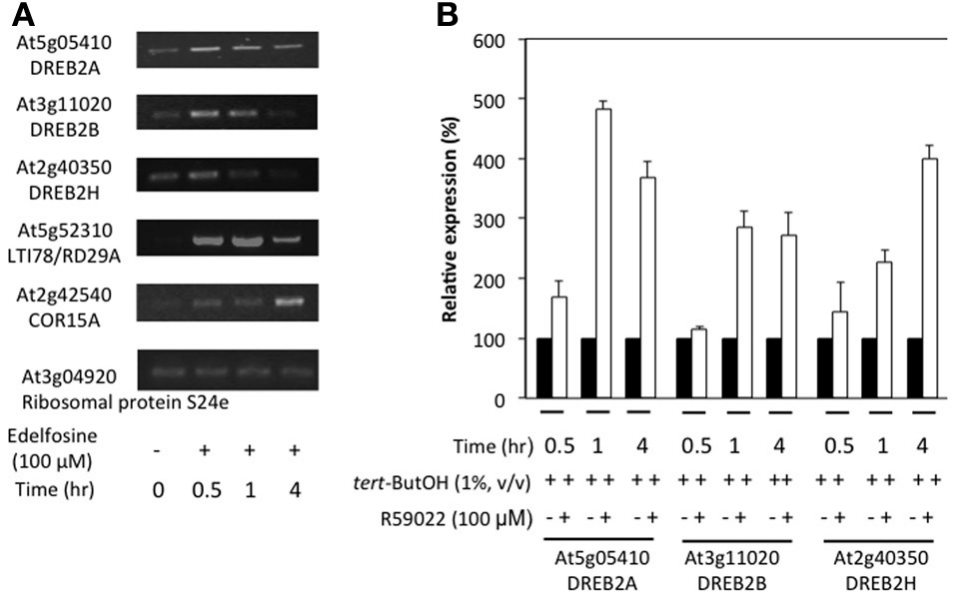
**Effects of inhibitors of PI-PLC or DGK on the expression levels of *DREB2* genes in seedlings**. Twelve-day old plants grown in liquid medium under continuous light were incubated with inhibitors and harvested at desired times. **(A)** Plants were treated with 100 M edelfosine. Transcript levels were estimated by reverse transcriptase-PCR, using an appropriate number of cycles. **(B)** Plants were treated with 100 M R59022. Transcript level were measured by qPCR, and expressed as % of the level in solvent treated plants at the desired time.

In order to establish whether the inhibitor effect is also evident in seedling tissues, 2-week old plants were treated with edelfosine (Figure 6A) or R59022 (Figure 6B). The inhibitors of PA production by PI-PLC pathway induced a clear expression of *DREB2A*, *DREB2B*, and *DREB2H* in the seedlings, and also of 2 DREB2-target genes, *LTI78* and *COR15A* (Figure 6A and Supplemental Figure S4).

### Is inhibition of phospholipase D (PLDs) overlapping inhibition of PLC pathway?

PA can be produced by another signaling pathway, namely PLDs. These enzymes hydrolyze structural lipids such as PC and PE into PA and a free alcohol. The question that rises is whether the genes described above as dependent on the level of PA originating from the PLC/DGK pathway for their expression, are also controlled by the PA from the PLDs.

PLD-dependent genes were revealed by comparing expression levels in presence of *n*-butanol (*n*-ButOH) and *tert*-butanol (*tert*-ButOH). Only the primary alcohol is a substrate of PLDs and leads to the formation of phosphatidyl-alcohol to the detriment of PA. We identified 1252 genes induced and 1304 genes repressed by *n*-ButOH *vs*. *tert*-ButOH (Supplemental Table S1I). We compared these lists to those obtained with R59022 or edelfosine. Only 111 genes had their expression altered by both *n*-ButOH and R59022. Comparing the contingency tables, it appeared that there was an over-representation (4-fold for genes induced by both molecules and 6-fold for repressed genes) of the 76 genes for which *n*-ButOH and R59022 had a similar effect. However, the contingency table also shows an over representation, even though to a lesser extent (2 to 3-times), of genes for which R59022 and *n*-ButOH had an opposite effect (35 genes; Table 4A). A Venn diagram representing the overlaps between gene regulated by *n*-ButOH and R59022 is in Supplemental Figure S1F.

**Table 4:** **Contingency table of the expression of genes responsive to *n*-ButOH as compared to the response to R59022 (A) or to edelfosine (B)**.

**Observed (Theoretical) ratio**				**Total**
	**Edelfosine > control**	**Edelfosine <> control**	**Edelfosine < control**	
**A**				
*n*-ButOH > *tert*-ButOH	91	1077	82	1250
	(21.59)	(1201.68)	(26.73)	
	**4.21**	**0.90**	**3.06**	
*n*-ButOH <> *tert*-ButOH	277	20531	268	21076
	(364.00)	(20261.26)	(450.75)	
	**0.76**	**1.01**	**0.59**	
*n*-ButOH < *tert*-ButOH	39	1047	154	1240
	(21.42)	(1192.06)	(26.52)	
	**1.86**	**0.88**	**5.81**	
Total	407	22655	504	23566
	**R59022 > control**	**R59022 <> control**	**R59022 < control**	
**B**				
*n*-ButOH > *tert*-ButOH	37	1248	17	1302
	(9.53)	(1286.18)	(6.30)	
	**3.88**	**0.97**	**2.70**	
*n*-ButOH <> *tert*-ButOH	122	21458	61	21641
	(158.32)	(21378.02)	(104.65)	
	**0.77**	**1.14**	**0.58**	
*n*-ButOH < *tert*-ButOH	18	1194	39	1251
	(9.15)	(1235.80)	(6.05)	
	**1.97**	**0.97**	**6.45**	
Total	177	23900	117	24194

*For each category, the number of observed genes can be compared to that of theoretical genes, indicated between brackets, considering the expression of genes to the 2 molecules, separately, are independent. The ratio “number of observed genes” vs. “theoretical number” is in bold*.

**Figure 7:**
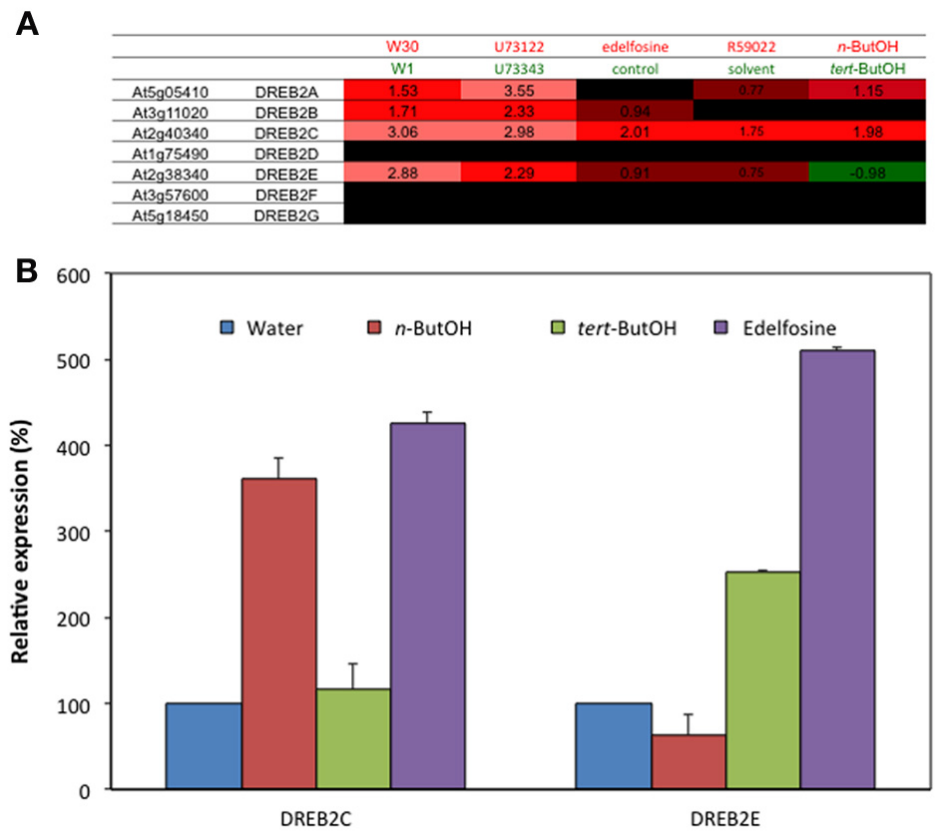
**Effects of *n*-ButOH on the expression levels of some *DREB2* genes in suspension cells**. **(A)** Expression level of *DREB2* genes extracted from microarray data. *DREB2H* is not present in the CATMA chip. **(B)** Transcript levels of *DREB2C* and *DREB2E* were quantified by qPCR and expressed as % of the level in control cells. *n* = 3.

In the case of edelfosine, 366 genes were affected by both treatments. Those for which the effects were similar (245 genes) were over-represented 4 to 6-times, and again those for which the effects were opposite (121 genes) were also over-represented (2–3 times; Table 4B). A Venn diagram representing the overlaps between gene regulated by *n*-ButOH and edelfosine is in Supplemental Figure S1G. The fact that basal PLD and basal PLC pathways do not necessarily control gene expression the same way is well-illustrated by *DREB2* genes. Our microarray data suggest that while *DREB2A*, *DREB2C*, and *DREB2E* are repressed by basal PLC coupled to DGK (induced by U73122 and R59022), *DREB2A*, and *DREB2C* were repressed by basal PLD activity whereas *DREB2E* was stimulated (Figure 7A). Using new set of RNAs the sensitivity to the inhibition of PI-LC and of PLD-produced PA was confirmed by qPCR for *DREB2C* and *DREB2E* genes (Figure 7B).

## Discussion

A pharmacological approach has allowed us to identify genes whose transcript levels are regulated by a basal PI-PLC activity in resting suspension cells. Edelfosine is a water-soluble molecule that has been shown to inhibit PI-PLC *in vitro* (Powis et al., 1992). It is been widely used to inhibit PI-PLC *in vivo*, either in animals or plants (Strassheim et al., 2000; Vergnolle et al., 2005; Thyagarajan et al., 2009). However, effects other than action on PI-PLC have been reported. Due to its structure close to lyso-PC, it could affect membrane structure (Wiese et al., 2000). Besides, edelfosine blocks PC biosynthesis (van der Luit et al., 2002), probably by acting on CTP:cholinephosphate cytidylyltransferase (Vogler et al., 1996). This is why it was important to cross the transcriptome data with those obtained with another PI-PLC inhibitor. U7322 is an aminosteroid that—till recently—was considered and used as the archetypal PI-PLC activity inhibitor. It has indeed been shown to inhibit *in vitro* PI-PLCs either from animals (Bleasdale and Fisher, 1993; Wilsher et al., 2007) or from plants (Takahashi et al., 2001; Helling et al., 2006). It is widely used in animals to study PI-PLC roles (Romano and Lograno, 2013; Zhong et al., 2013). In plants, U73122 was shown to lead to an increase of PI-PLC substrates in tobacco plasma membrane (Van Leeuwen et al., 2007) and in pollen tube tip (Helling et al., 2006). It was shown to diminish PI-PLC *in vivo* activity during osmotic stress (Takahashi et al., 2001; Parre et al., 2007; Ghars et al., 2012), heat stress (Liu et al., 2006), cold stress (Vergnolle et al., 2005), gravistimulation (Perera et al., 2001), and to diminish calcium oscillation in guard cells in response to ABA (Staxen et al., 1999). Recently, it was used to show PI-PLC involvement in phototropic gene expression (Salinas-Mondragon et al., 2010) and in blue light-mediated chloroplast movements (Aggarwal et al., 2013). It was also shown to inhibit a basal PI-PLC activity in *Coffea arabica* cells (Ramos-Díaz et al., 2007). However, even if the role of U73122 as a PI-PLC inhibitor is well-documented, it is believed to act as a general alkylating agent. Because side effects might result from protein alkylation (Mogami et al., 1997; Horowitz et al., 2005) it has been recommended to preferentially use edelfosine over U73122 to inhibit PI-PLC (Wong et al., 2007; Kelm et al., 2010). We suggest that comparing the effects of these 2 PI-PLC inhibitors, because of their different chemistries, is an appropriate way to detect what is really attributable to PI-PLC.

Among genes whose expression was affected by the inhibitors, we found an under representation of genes for which the inhibitors had opposite effects (3 genes), concomitantly with an over representation of genes for which they had the same effect (128 genes induced, 122 genes inhibited). One might consider that these numbers are not high. However, taking into account that the transcriptome data were normalized using *Bonferonni* correction, the significance thresholds are stringent. With a less stringent correction [Benjamini-Hochberg (BH) correction] just for U73122 analysis, we found 247 genes inhibited and 213 genes induced by both inhibitors. If the BH correction was also used for edelfosine analysis, it led to 293 genes induced and 323 genes repressed by both inhibitors. Rather than the absolute number of genes in each category, it is the enrichment ratio as shown in the contingency tables that is pertinent. Indeed, the genes induced by U73122 or edelfosine, and the genes repressed by those chemicals, are 10- and 16-fold more abundant than expected in a random distribution. The strong correlation of edelfosine- and U3122-altered genes made us consider those genes as being PI-PLC controlled.

PI-PLC action results in the production of InsP_3_, and of DAG that can be phosphorylated into PA. It also results in a decrease of its substrates, the phosphorylated phosphatidylinositol (the *so*-called phosphoinositides). Indeed, in response to cold, we monitored a decrease of PI4P/PI(4,5)P_2_ as PA increased due to PI-PLC activation in Arabidopsis suspension cells (Ruelland et al., 2002). Van Leeuwen et al. (2007) could visualize PI(4,5)P_2_ in plasma membrane of tobacco BY-2 cells only when PI-PLC was inhibited by U73122, showing that (i) a basal PI-PLC activity exists in plants, (ii) this basal PI-PLC activity diminishes phosphoinositide level. Since phosphoinositides can bind proteins (Van Leeuwen et al., 2004; Delage et al., 2013), it is possible that PI-PLC pathway controls gene expression by the increase of its products or by the decrease of its substrates. We recently demonstrated that PI-PLC substrates are provided by wortmannin-sensitive type III-PIKs (Delage et al., 2012). Inhibiting type III-PI4Ks leads to a decrease of PI-PLC products due to a decrease of PI-PLC substrates. On the contrary, inhibiting PI-PLCs would lead to an increase of PI-PLC substrates. Therefore, if the action of PI-PLC on gene expression is mediated by a PI-PLC product, inhibiting PI-PLCs or type III-PI4Ks will lead to a similar effect on the gene response. On the contrary, if the action of PI-PLC is through a control by its substrates, inhibiting type III-PI4Ks and inhibiting PI-PLCs should lead to opposite effects (working model in Supplemental Figures S2B–D). We compared the edelfosine transcriptome data with that obtained with 30 M wortmannin (Krinke et al., 2007). We detected 596 genes whose expression was similarly affected by edelfosine and wortmannin, this being at least 10-fold more than expected in case of a random distribution. This represented 65% of all edelfosine-regulated genes, a percentage that was certainly underestimated due to threshold considerations (see above). On the contrary, only 22 genes were identified for which edelfosine and wortmannin had opposite effects. This is less than what would be expected considering the inhibitor treatments were independent. The same results arise when crossing wortmannin data with U73122 ones. This strongly supports the notion that basal PI-PLCs act through their products. Here, also, it is necessary to consider wortmannin specificity. We have demonstrated that W30 inhibits *in vitro* PI4Ks from Arabidopsis membranes (Delage et al., 2012). It also inhibits PI4Ks *in vivo* (Krinke et al., 2007). Yet, we cannot rule out that it also inhibits protein kinases, such as ataxia-telangiectasia-mutated protein (Sarkaria et al., 1998). However, the strong overlap between W30 and edelfosine (or U73122) effects is in favor of an action on the same pathway, and of basal PI-PLC acting through its products.

The basal PI-PLCs produce InsP_3_ and its phosphorylated derivatives, and DAG and its phosphorylated derivative, PA. We tested the implication of PA by inhibiting DGK with R59022. This molecule was shown to inhibit *in vitro* DGK either from plants (Lundberg and Sommarin, 1992; Gómez-Merino et al., 2005; Vaultier et al., 2008) or from animals (Lundberg and Sommarin, 1992; Matowe and Ginsberg, 1996; Jiang et al., 2000; Gómez-Merino et al., 2005). The *in vivo* synthesis of cold-induced PA was reduced by treatment with R59022 in Arabidopsis suspension cells (Ruelland et al., 2002); R59022 enhanced phytoalexin accumulation in pea (*Pisum sativum*) treated with fungal elicitor (Toyoda et al., 2000). As gene expression was affected by this treatment, this implies that PA is transducing basal PI-PLC action. Many of the genes whose expression was altered by the DGK inhibitor had their expression altered in a similar way by edelfosine. Their number was 24-fold more than expected if the actions of the inhibitors were independent. Therefore, it shows that DGKs, acting downstream of PLCs, play a significant role in cell physiology. Documented roles for DGK are still scarce. Arabidopsis seedlings grown for 3 weeks in the presence of R59022 had a reduced primary root elongation, suggesting a role for DGKs in root growth (Gómez-Merino et al., 2005). Rice protoplasts expressing RNAi-silencing constructs targeting multiple *DGK*s are impaired in the expression of *PR1* in response to xylanase, and in the expression of *CIPK15* in response to NaCl (Ge et al., 2012).

Interestingly, amongst the genes induced by R59022 are most of *DREB2* genes. This is true for both suspension cells and in seedlings. These genes are also induced after inhibiting PI-PLCs. DREB2 proteins are transcription factors with an AP2/ERF DNA-binding domain. In Arabidopsis, there are 8 *DREB2* genes, named *DREB2A* to *DREB2H* (Ruelland et al., 2009). *DREB2A* and *DREB2B* are highly induced by drought, NaCl, or heat, while poor induction is seen in response to cold or ABA (Nakashima et al., 2000; Sakuma et al., 2006). Expression of *DREB2C*, *DREB2D*, and *DREB2F* is slightly induced by high salt treatments in leaves, but much less than that of *DREB2A* and *DREB2B*. Expression of *DREB2E* is slightly induced in roots only by ABA. No conditions have yet been shown to result in the induction of *DREB2G* and *DREB2H* (Sakuma et al., 2002). Here, *DREB2H* was clearly induced by inhibiting DGKs or PI-PLCs. *DREB2A* is a major transcription factor governing gene expression during osmotic stress in an ABA-independent pathway (Nakashima et al., 2009). *DREB2A* has also a major role in heat response by inducing *HsfA3* promoter (Sakuma et al., 2006; Schramm et al., 2008; Yoshida et al., 2008). The transcriptomes of the heat or osmotic stress responses are indeed the stress-microarray experiments with the highest correlation rates to edelfosine-microarray or R590022-microarray. Accordingly, the motif of the *cis*-elements bound by DREB2 transcription factors are over-represented in genes induced by edelfosine and W30 (i.e., genes induced by inhibiting the production of PI-PLC products).

Our data thus confirm a coupling between type III-PI4Ks, PI-PLCs, and DGKs. This coupling leads to the basal inhibition of most *DREB2* gene expression. In Arabidopsis, all these enzymes are encoded by multigenic families. However, the pharmacological approach taken here does not give information about the isoforms involved in this coupling. The mode of inhibition of type III-PI4Ks by wortmannin is known: it binds irreversibly to a lysine in the active site of the lipid kinases (Wymann et al., 1996). This lysine being conserved in the 3 Arabidopsis type III-PI4Ks (Lys-1772, Lys-862, and Lys-859 for AtPI4K1, AtPI4K1, and AtPI4K2, respectively) wortmannin is not likely to be able to discriminate between them. In response to cold, we have shown that the 3 type III-PI4Ks indeed participated in the synthesis of substrates of PI-PLCs (Delage et al., 2012). This could also be the case in resting conditions. The plant PI-PLCs being all very similar, and structurally related to Mammalian PI-PLC, it is very unlikely that U73122 or edelfosine would discriminate between them. The inhibiting role of U73122 is clearly established, but its exact mode of action is not documented. As already mentioned, it might act as an alkylating agent (Mogami et al., 1997; Horowitz et al., 2005), but the target residues on PI-PLC are not known. Therefore, it is not possible to consider a specific U73122-inhibition on some Arabidopsis PI-PLC isoforms. Edelfosine, an alkyl-lysophosphatidylcholine, is likely to act by competitive inhibition, but here again, the specificity within PI-PLC family is not known. Thus, U73122, edelfosine, R59022, or R59949, are not likely to discriminate between their respective targets that are also functionally redundant, as reported for PI-PLC (Pokotylo et al., 2014) and DGK (Arisz et al., 2013). All PI-PLCs or DGKs present in basal suspension cells or seedlings likely participate in the negative control of *DREB2* gene.

It is possible that a basal PLD activity occurs in cells, in this way also generating PA although composed of different molecular species (Rainteau et al., 2012). If this is so, does its inhibition induce the expression of *DREB2* genes? In fact, different responses exist: induction, repression or no effect. So PLD-generated and PLC/DGK-generated PAs are not equivalent. Besides the differences in composition, the environment of PA could differ with the membrane where it appears according to the location of the isoform responsible for its synthesis.

Some published data show that PI-PLCs have a role in controlling a basal process. Incubation of *Thellungiella salsuginea* seedlings with U73122 did lead to an increase in cellular proline level (Ghars et al., 2012). Which PI-PLC product was involved is not known. Resting InsP_3_ levels were monitored in tobacco cells or Arabidopsis plants, and the expression of mammalian inositol polyphosphate 5-phosphatase activity (that specifically hydrolyses soluble inositol phosphates) led to a reduction of the basal InsP_3_ levels (Perera et al., 2002, 2008). Those transgenic plants were characterized by the up-regulation of *DREB2A* gene and some of its target genes (Perera et al., 2008). This data would suggest that *DREB2A* is constitutively repressed by a basal PI-PLC, *via* its production of soluble phosphorylated inositols. Both our study and that of Perera et al. (2008) suggest that there is a basal PI-PLC negative regulation of *DREB2A* expression. Both the lipid and the soluble mediators produced by this basal PI-PLC seem to participate in *DREB2* down regulation. Deciphering the respective roles of soluble inositols and PA, either additive or opposite, in the PI-PLC triggered responses, requires further investigation.

Does the fact that PI-PLC/DGK exerts a negative control on basal *DREB2A* imply that in order to get an increased *DREB2A* expression in response to stresses, PI-PLC activity has to be reduced? In fact, far from being associated with PI-PLC activity inhibition, osmotic stresses are associated with an increase in PI-PLC activity. InsP_3_ has been shown to increase with salt stress and mannitol (osmotic) stress in Arabidopsis suspension cells (Takahashi et al., 2001) and Arabidopsis seedlings (Parre et al., 2007). However, the exact role of PI-PLCs in response to osmotic stress is not fully understood. In Arabidopsis suspension cells, the *DREB2A* induction by mannitol did not seem to be inhibited by neomycin, a molecule that chelates phosphoinositides and therefore inhibits the PI-PLC pathway (Takahashi et al., 2001). That would suggest that PI-PLC activation might not be a major actor of response to osmotic stress, or at least of the *DREB2A* induction. Indeed osmotic stresses activate pathway others than the PI-PLC one. In Arabidopsis, osmotic stress is associated with an increased PI(4,5)P_2_ content (Takahashi et al., 2001; König et al., 2007). This is possible that the PI(4,5)P_2_ producing enzymes are more activated than the degrading enzymes (PI-PLC) during stress.

The apparent contradiction whereby basal PI-PLC activity represses the expression of stress-associated genes (e.g., *DREB2*s), while stress-activated PI-PLCs stimulate their expression, only exists considering that PI-PLCs use the same modes of action in basal and stress conditions. A major difference between these conditions will be the content in both PI-PLC substrates and products. If we consider solely a PI-PLC activation over its basal activity level, it would lead to a decrease in phosphoinositides and an increase in products (Supplemental Figure S2E). Thus, there would be more PA, for instance, present in stressed cells than in resting ones. This should affect PA interactions with its target peptides: in resting conditions, only the high affinity ones would be involved. It is possible that these targets negatively regulate *DREB2* gene responses. In stress conditions, PA at high concentrations could interact with targets of lower affinity. These targets may have opposite effects compared to the high affinity targets: they may positively regulate *DREB2A* expression. Moreover, some stress conditions have been associated with a situation where phosphoinositide level is increased concomitantly with the PI-PLC activation (Supplemental Figure S2F). PI(4,5)P_2_ could be one of the active molecules during the response to some stresses, positively regulating *DREB2* gene expression. Interestingly, PI(4,5)P_2_ increase has been reported in response to osmotic stresses, but also to heat stress, two stresses that induce *DREB2* expression.

Some PLDs also participate in osmotic stress responses (Hong et al., 2008) and thus they might play a role more important than that of PI-PLC. Indeed, the mannitol-trigerred proline accumulation in *Thellungiella salsuginea* was moderately inhibited by U73122, while it was strongly inhibited by *n*-ButOH (Ghars et al., 2012). Clearly, the role of PI-PLC on *DREB2* gene expression in response to osmotic stress should be further investigated. What appears is that several cellular processes (such as *DREB2* expression, or proline accumulation) are actively regulated in both stress and basal conditions. However, signaling pathways in “stress” conditions are not the simple “reversion” or, on the contrary, “enhancement” of the ones active in basal conditions. Understanding plant physiology implies to understand signaling or regulation both in basal conditions and in stress activated conditions.

Finally, it has been shown that genes orthologous to Arabidopsis *DREB* genes are induced during desiccation in resurrection plants, i.e., plants that are able to survive the loss of more than 90% of their water content loss (Mundree et al., 2002; Garwe et al., 2003). Our data opens the question as to the role of lipid signaling in controlling the expression of *DREB* genes, in basal states and in stressed states. Is lipid signaling also involved in basal and stress induced regulation of *DREB2* genes in resurrection plants? That is, are the lipid signaling pathways active during initial drying of water loss up to 40%, (Morse et al., 2011), which is akin to dehydration and furthermore, are they also active during the more severe stages of water loss (of up to 95% of total water content), akin to desiccation? And are the roles during dehydration and desiccation similar, in particular in the control of gene expression? The data we present in this article illustrate the complexity of the regulation of *DREB2* genes, and the prominent role of PI-PLC pathway in this regulation. An exciting prospect is now to investigate the role of this signaling pathway in plants not only able to survive drought, but also desiccation.

## Materials and methods

### Cell culture and pharmacological treatments

*Arabidopsis thaliana* Col-0 suspension cells were cultivated as in (Krinke et al., 2009). Experiments were performed on 5-d-old cultures. Edelfosine (20 mM in water), R59022, R59949 (10 mM in *tert*-ButOH), and wortmaninn (6 mM in methanol) were used as stock solutions. *Arabidopsis thaliana* seeds were sterilized as in (Delage et al., 2012). Five to ten seeds were sown in wells of 24-well plates filled with 400 μL basic half-strength Murashige and Skoog (MS) medium, pH 5.7, supplemented with 5 g.L^−1^ MES, 5 g.L^−1^ sucrose. After 3d at 4°C, plates were transferred at 22°C, under white light 100 E.m^−2^.s^−1^, 16 h day/8 h night. Experiments were performed on 2 week-old seedlings.

### Transcriptome studies

Microarray analysis was carried out at the Unité de Recherche en Génomique Végétale (Evry, France), using CATMA arrays containing 31776 gene-specific tags corresponding to 22089 genes from Arabidopsis (Crowe et al., 2003; Hilson et al., 2004). Two independent biological replicates were made. For each biological repetition and each point, samples were obtained by pooling RNAs from 3 independent experiments. RNAs were prepared as described in Krinke et al. (2009). For each comparison, one technical replicate with fluorochrome reversal was performed for each biological replicate (i.e., four hybridizations per comparison). Labeling of cRNAs with Cy3-dUTP or Cy5-dUTP (Perkin-Elmer-NEN Life Science Products), hybridization to slides, and their scanning were performed as described in Lurin et al. (2004).

### Statistical analysis of microarray data

Experiments were designed with the statistics group of the Unité de Recherche en Génomique Végétale. For each array, the raw data comprised the logarithm of median feature pixel intensity at wavelengths 635 nm (red) and 532 nm (green) and no background was subtracted. An array-by-array normalization was performed to remove systematic biases. First, spots considered badly formed features were excluded. Then a global intensity-dependent normalization using the loess procedure (Yang et al., 2002) was performed to correct the dye bias. Finally, for each block, the log-ratio median calculated over the values for the entire block was subtracted from each individual log-ratio value to correct print tip effects. Differential analysis was based on the log ratios averaged on the dye-swap: The technical replicates were averaged to get one log-ratio per biological replicate and these values were used to perform a paired *t*-test. A trimmed variance was calculated from spots which did not display extreme variance (Gagnot et al., 2008). The raw *p*-values were adjusted by the Bonferroni method, which controls the Family Wise Error Rate in order to keep a strong control of the false positives in a multiple-comparison context. We considered as being differentially expressed the probes with a Bonferroni *p*-value < 0.05.

### Semiquantitative RT-PCR analysis and qPCR analysis

For semiquantitative RT-PCR, 1 μ g of total RNA was treated with DNase I (Sigma-Aldrich) and reverse transcribed using the Omniscript reverse transcriptase kit from Qiagen and oligo(dT)_15_ primers according to the supplier's instructions. An equivalent of 40 ng of total RNA was amplified with 0.6 μ M gene-specific primer pairs. The gene encoding a 40S ribosomal protein S24 (At3g04920) was used as a housekeeping gene. Annealing temperature was 53°C for all primer pairs. A suitable number of PCR cycles were used for each primer pair. PCR was separated on horizontal electrophoresis in 1% (w/v) agarose gels, 0.5× TBE buffer. Quantitative PCR (qPCR) was realized as in Delage et al. (2012), with a Eppendorf Mastercycler® gradient thermal cycler. Threshold cycles (C_T_) for each sample were determines with Ependorf Realpex 2.0 software. Expression for each gene of interest was normalized to the expression of the housekeeping gene, Cyt b_5_ (At5g53560). Primers specific for each gene were used.

### Motif analysis

Promoters (up to −1000 bp, without overlaps with other genes, with exclusion of 5′UTRs) were extracted from the database of The Arabidopsis Information Resource (TAIR; Rhee et al., 2003). Created lists of promoters were searched for overrepresented motifs ranging from 4 to 10 bp using SIFT software (Hudson and Quail, 2003) with a list of whole genome promoters (33062 promoters) as a reference (promoters regions up to −1000 bp, without overlaps with other genes, with exclusion of 5′UTRs). Overrepresented motifs (with a *P*-value above a threshold of 10^−5^, Chi-squared test) were subsequently aligned using ClustalX in order to cluster modules of the same motif. The motifs thus defined were then compared with those in PLACE (Higo et al., 1999), RARGE (Jirage et al., 2001), and AGRIS (Yilmaz et al., 2010) databases to search for related *cis*-elements and the closest motifs.

## Data sharing

Newly generated microarray data from this article were deposited at Gene Expression Omnibus (http://www.ncbi.nlm.nih.gov/geo/; accession no. GSE19850, GSE 35872 and GSE46941) and at CATdb (http://urgv.evry.inra.fr/CATdb/; Projects: RS09-04, AU10-12, AU13-01) according to the “Minimum Information About a Microarray Experiment” standards.

### Conflict of interest statement

The authors declare that the research was conducted in the absence of any commercial or financial relationships that could be construed as a potential conflict of interest.

## Supplementary material

Supplemental Table S1 **Genes whose basal expression is altered by pharmacological agents. (A)** List of genes whose expression is altered by edelfosine. **(B)** List of genes whose expression is altered by U73122 vs. U73343. **(C)** List of genes whose expression is altered the same way by U73122 vs. U73343 and edelfosine. **(D)** List of genes whose expression is altered by W30 vs. W1. **(E)** List of genes whose expression is altered the same way by W30 vs. W1 and edelfosine. **(F)** List of genes whose expression is altered by R59022. **(G)** List of genes whose expression is altered the same by R59022 and edelfosine. **(H)** List of genes whose expression is altered the same way by W30 vs. W1, edelfosine and R59022. **(I)** List of genes whose expression is altered by n-ButOH vs. tert-ButOH.

Supplemental Table S2 **Contingency tables. (A)** Contingency table of the expression of genes in response to U73122 and W30. **(B)** Contingency table of the expression of genes in response to edelfosine and R59022. **(C)** contingency table of the expression of genes in response to edelfosine and R59022 and W30.

Supplemental Table S3 **Genes whose expression is altered by edelfosine or R59022 have their expression altered in mutants overexpressing native or constitutively active form of DREB2A from Arabidopsis or *Glycine max*. (A)** Genes whose expression is altered by edelfosine. **(B)** Genes whose expression is altered by R59022. The expression values in mutants overexpressing native or constitutively active form of DREB2A from Arabidopsis or *Glycine max* are from Mizoi et al. (2013).

Supplemental Figure S1 **Venn diagrams. (A)** Venn diagram representation of the overlaps between genes regulated by U73122 or edelfosine. **(B)** Venn diagram representation of the overlaps between genes regulated by W30 or edelfosine (Edel). **(C)** Venn diagram representation of the overlaps between genes regulated by R59022 or edelfosine (Edel). **(D)** Venn diagram representation of the overlaps between genes induced by W30, or R59022 or edelfosine (Edel). **(E)** Venn diagram representation of the overlaps between genes repressed by W30, or R59022 or edelfosine (Edel). **(F)** Venn diagram representation of the overlaps between genes regulated by R59022 or *n*-ButOH (nBut). **(G)** Venn diagram representation of the overlaps between genes regulated by *n*-ButOH (nBut) and edelfosine (Edel). + indicates genes induced by the molecule, − indicates genes repressed by the molecule.

Supplemental Figure S2 **Working models of PI-PLC actions. (A)** In resting cells, basal PI-PLCs produce InsP3 that can be transformed into other phosphorylated inositols, and DAG that can be phosphorylated, into PA. Phosphorylated inositols and PA can activate transducing pathways that lead to DREB2 gene basal down-regulation. **(B)** Simplified version of **(A)**, to be used for comparison with other panels. **(C)** When the basal PI-PLCs are inhibited, this leads to a decrease in products, concomitantly with an increase in the substrates, the phosphorylated PI (named phosphoinositides). **(D)** The first committing enzymes phosphorylating PI into the phosphoinositides substrates of PI-PLCs are the type III-PI4K (Delage et al., 2012 and this article). Their inhibition by 30 μ M wortmannin leads to a decrease in phosphoinositides but also in PI-PLC products. **(E)** PI-PLC activation would lead to a decrease of the phosphoinositide pools, concomitantly with an increase of PI-PLC products. **(F)** In some stress situations, a PI-PLC has been reported to be activated, but associated with an increase of phosphoinositides, which can happen if the phosphoinositide-producing pathway is activated at a higher rate than the PI-PLC activation.

Supplemental Figure S3 **Classification of edelfosine- and W30-induced or repressed genes according to their biological processes**. For each biological process, the ratio to whole Arabidopsis set (genome) is shown. In gray bars are the categories not statistically different from the whole Arabidopsis set. Bars in bright colors are for the over-represented categories and that in shaded colors the under-represented ones.

Supplemental Figure S4 **Effects of R59022 on the expression levels of *DREB2* genes and target genes in seedlings**. Twelve-day old plants grown in liquid medium under continuous light were incubated with inhibitors and harvested at desired times. Transcript levels were estimated by reverse transcriptase-PCR, using an appropriate number of cycles.

## References

[B1] Aggarwal C. Labuz J. Gabryś H. (2013). Phosphoinositides play differential roles in regulating phototropin1- and phototropin2-mediated chloroplast movements in Arabidopsis. PLoS ONE 8:e55393 10.1371/journal.pone.0055393 23405144 PMC3566141

[B2] Arisz S. A. Testerink C. Munnik T. (2009). Plant PA signaling via diacylglycerol kinase. Biochim. Biophys. Acta 1791, 869–875 10.1016/j.bbalip.2009.04.006 19394438

[B3] Arisz S. A. van Wijk R. Roels W. Zhu J.-K. Haring M. A. Munnik T. (2013). Rapid phosphatidic acid accumulation in response to low temperature stress in Arabidopsis is generated through diacylglycerol kinase. Front. Plant Sci. 4:1 10.3389/fpls.2013.00001 23346092 PMC3551192

[B4] Baker S. S. Wilhelm K. S. Thomashow M. F. (1994). The 5'-region of *Arabidopsis thaliana* cor15a has cis-acting elements that confer cold-, drought- and ABA-regulated gene expression. Plant Mol. Biol. 24, 701–713 10.1007/BF00029852 8193295

[B5] Bargmann B. O. Munnik T. (2006). The role of phospholipase D in plant stress responses. Curr. Opin. Plant Biol. 9, 515–522 10.1016/j.pbi.2006.07.011 16877031

[B6] Bleasdale J. E. Fisher S. K. (1993). Use of U-73122 as an inhibitor of phospholipase C-dependent processes. Neuroprotocols 3, 125–133 10.1006/ncmn.1993.1046

[B7] Boss W. F. Sederoff H. W. Im Y. J. Moran N. Grunden A. M. Perera I. Y. (2010). Basal signaling regulates plant growth and development. Plant Physiol. 154, 439–443 10.1104/pp.110.161232 20921159 PMC2948987

[B8] Chan C. S. Guo L. Shih M. C. (2001). Promoter analysis of the nuclear gene encoding the chloroplast glyceraldehyde-3-phosphate dehydrogenase B subunit of *Arabidopsis thaliana*. Plant Mol. Biol. 46, 131–141 10.1023/A:1010602031070 11442054

[B9] Crowe M. L. Serizet C. Thareau V. Aubourg S. Rouzé P. Hilson P. (2003). CATMA: a complete Arabidopsis GST database. Nucleic Acids Res. 31, 156–158 10.1093/nar/gkg071 12519971 PMC165518

[B10] Delage E. Puyaubert J. Zachowski A. Ruelland E. (2013). Signal transduction pathways involving phosphatidylinositol 4-phosphate and phosphatidylinositol 4, 5-bisphosphate: convergences and divergences among eukaryotic kingdoms. Prog. Lipid Res. 52, 1–14 10.1016/j.plipres.2012.08.003 22981911

[B11] Delage E. Ruelland E. Guillas I. Zachowski A. Puyaubert J. (2012). Arabidopsis type-III phosphatidylinositol 4-kinases β 1 and β 2 are upstream of the phospholipase C pathway triggered by cold exposure. Plant Cell Physiol. 53, 565–576 10.1093/pcp/pcs011 22318862

[B12] Gagnot S. Tamby J.-P. Martin-Magniette M.-L. Bitton F. Taconnat L. Balzergue S. (2008). CATdb: a public access to Arabidopsis transcriptome data from the URGV-CATMA platform. Nucleic Acids Res. 36, D986–D990 10.1093/nar/gkm757 17940091 PMC2238931

[B13] Garwe D. Thomson J. A. Mundree S. G. (2003). Molecular characterization of XVSAP1, a stress-responsive gene from the resurrection plant *Xerophyta viscosa* Baker1. J. Exp. Bot. 54, 191–201 10.1093/jxb/erg013 12493847

[B14] Ge H. Chen C. Jing W. Zhang Q. Wang H. Wang R. (2012). The rice diacylglycerol kinase family: functional analysis using transient RNA interference. Front. Plant Sci. 3:60 10.3389/fpls.2012.00060 22639654 PMC3355625

[B15] Ghars M. A. Richard L. Lefebvre-De Vos D. Leprince A.-S. Parre E. Bordenave M. (2012). Phospholipases C and D modulate proline accumulation in *Thellungiella halophila/salsuginea* differently according to the severity of salt or hyperosmotic stress. Plant Cell Physiol. 53, 183–192 10.1104/pp.106.095281 22121247

[B16] Giraud E. Ho L. H. M. Clifton R. Carroll A. Estavillo G. Tan Y.-F. (2008). The absence of ALTERNATIVE OXIDASE1a in Arabidopsis results in acute sensitivity to combined light and drought stress. Plant Physiol. 147, 595–610 10.1104/pp.107.115121 18424626 PMC2409015

[B17] Goldsbrough A. P. Albrecht H. Stratford R. (1993). Salicylic acid-inducible binding of a tobacco nuclear protein to a 10 bp sequence which is highly conserved amongst stress-inducible genes. Plant J. 3, 563–571 10.1046/j.1365-313X.1993.03040563.x 8220463

[B18] Gómez-Merino F. C. Arana-Ceballos F. A. Trejo-Téllez L. I. Skirycz A. Brearley C. A. Dörmann P. (2005). Arabidopsis AtDGK7, the smallest member of plant diacylglycerol kinases (DGKs), displays unique biochemical features and saturates at low substrate concentration: the DGK inhibitor R59022 differentially affects AtDGK2 and AtDGK7 activity *in vitro* and alters plant growth and development. J. Biol. Chem. 280, 34888–34899 10.1074/jbc.M506859200 16081412

[B19] Gómez-Porras J. L. Riaño-Pachón D. M. Dreyer I. Mayer J. E. Mueller-Roeber B. (2007). Genome-wide analysis of ABA-responsive elements ABRE and CE3 reveals divergent patterns in Arabidopsis and rice. BMC Genomics 8:260 10.1186/1471-2164-8-260 17672917 PMC2000901

[B20] Helling D. Possart A. Cottier S. Klahre U. Kost B. (2006). Pollen tube tip growth depends on plasma membrane polarization mediated by tobacco PLC3 activity and endocytic membrane recycling. Plant Cell 18, 3519–3534 10.1105/tpc.106.047373 17172355 PMC1785407

[B21] Higo K. Ugawa Y. Iwamoto M. Korenaga T. (1999). Plant cis-acting regulatory DNA elements (PLACE) database: 1999. Nucleic Acids Res. 27, 297–300 10.1093/nar/27.1.297 9847208 PMC148163

[B22] Hilson P. Allemeersch J. Altmann T. Aubourg S. Avon A. Beynon J. (2004). Versatile gene-specific sequence tags for Arabidopsis functional genomics: transcript profiling and reverse genetics applications. Genome Res. 14, 2176–2189 10.1101/gr.2544504 15489341 PMC528935

[B23] Himmelbach A. Hoffmann T. Leube M. Höhener B. Grill E. (2002). Homeodomain protein ATHB6 is a target of the protein phosphatase ABI1 and regulates hormone responses in Arabidopsis. EMBO J. 21, 3029–3038 10.1093/emboj/cdf316 12065416 PMC126069

[B24] Hong Y. Pan X. Welti R. Wang X. (2008). Phospholipase Dα 3 is involved in the hyperosmotic response in Arabidopsis. Plant Cell 20, 803–816 10.1105/tpc.107.056390 18364466 PMC2329935

[B25] Horowitz L. F. Hirdes W. Suh B.-C. Hilgemann D. W. Mackie K. Hille B. (2005). Phospholipase C in living cells activation, inhibition, Ca2+ requirement, and Regulation of M current. J. Gen. Physiol. 126, 243–262 10.1085/jgp.200509309 16129772 PMC2266577

[B26] Hruz T. Laule O. Szabo G. Wessendorp F. Bleuler S. Oertle L. (2008). Genevestigator v3: a reference expression database for the meta-analysis of transcriptomes. Adv. Bioinformatics 2008:420747 10.1155/2008/420747 19956698 PMC2777001

[B27] Hudson M. E. Quail P. H. (2003). Identification of promoter motifs involved in the network of phytochrome A-regulated gene expression by combined analysis of genomic sequence and microarray data. Plant Physiol. 133, 1605–1616 10.1104/pp.103.030437 14681527 PMC300717

[B28] Hunt L. Otterhag L. Lee J. C. Lasheen T. Hunt J. Seki M. (2004). Gene−specific expression and calcium activation of *Arabidopsis thaliana* phospholipase C isoforms. New Phytol. 162, 643–654 10.1111/j.1469-8137.2004.01069.x 33873763

[B29] Janda M. Planchais S. Djafi N. Martinec J. Burketova L. Valentova O. (2013). Phosphoglycerolipids are master players in plant hormone signal transduction. Plant Cell Rep. 32, 839–851 10.1007/s00299-013-1399-0 23471417

[B30] Jiang Y. Sakane F. Kanoh H. Walsh J. P. (2000). Selectivity of the diacylglycerol kinase inhibitor 3-{2-(4-[bis-(4-fluorophenyl)methylene]-1-piperidinyl) ethyl}-2,3-dihydro-2-thioxo-4(1H)quinazolinone (R59949) among diacylglycerol kinase subtypes. Biochem. Pharmacol. 59, 763–772 10.1016/S0006-295200395-0 10718334

[B31] Jirage D. Zhou N. Cooper B. Clarke J. D. Dong X. Glazebrook J. (2001). Constitutive salicylic acid-dependent signaling in cpr1 and cpr6 mutants requires PAD4. Plant J. 26, 395–407 10.1046/j.1365-313X.2001.2641040.x 11439127

[B32] Jung C. Seo J. S. Han S. W. Koo Y. J. Kim C. H. Song S. I. (2008). Overexpression of AtMYB44 enhances stomatal closure to confer abiotic stress tolerance in transgenic Arabidopsis. Plant Physiol. 146, 623–635 10.1104/pp.107.110981 18162593 PMC2245844

[B33] Kelm M. K. Weinberg R. J. Criswell H. E. Breese G. R. (2010). The PLC/IP3R/PKC pathway is required for ethanol-enhanced GABA release. Neuropharmacology 58, 1179–1186 10.1016/j.neuropharm.2010.02.018 20206640 PMC2849882

[B34] König S. Mosblech A. Heilmann I. (2007). Stress-inducible and constitutive phosphoinositide pools have distinctive fatty acid patterns in *Arabidopsis thaliana*. FASEB J. 21, 1958–1967 10.1096/fj.06-7887com 17327357

[B35] Krinke O. Flemr M. Vergnolle C. Collin S. Renou J.-P. Taconnat L. (2009). Phospholipase D activation is an early component of the salicylic acid signaling pathway in Arabidopsis cell suspensions. Plant Physiol. 150, 424–436 10.1104/pp.108.133595 19304931 PMC2675726

[B36] Krinke O. Ruelland E. Valentová O. Vergnolle C. Renou J.-P. Taconnat L. (2007). Phosphatidylinositol 4-kinase activation is an early response to salicylic acid in Arabidopsis suspension cells. Plant Physiol. 144, 1347–1359 10.1104/pp.107.100842 17496105 PMC1914138

[B37] Laxalt A. M. Raho N. Have A. T. Lamattina L. (2007). Nitric oxide is critical for inducing phosphatidic acid accumulation in xylanase-elicited tomato cells. J. Biol. Chem. 282, 21160–21168 10.1074/jbc.M701212200 17491015

[B38] Liu H. T. Gao F. Cui S. J. Han J. L. Sun D. Y. Zhou R. G. (2006). Primary evidence for involvement of IP3 in heat-shock signal transduction in Arabidopsis. Cell Res. 16, 394–400 10.1038/sj.cr.7310051 16617335

[B39] Lundberg G. A. Sommarin M. (1992). Diacylglycerol kinase in plasma membranes from wheat. Biochim. Biophys. Acta 1123, 177–183 10.1016/0005-276090109-9 1310876

[B40] Lurin C. Andrés C. Aubourg S. Bellaoui M. Bitton F. Bruyère C. (2004). Genome-wide analysis of Arabidopsis pentatricopeptide repeat proteins reveals their essential role in organelle biogenesis. Plant Cell 16, 2089–2103 10.1105/tpc.104.022236 15269332 PMC519200

[B41] Matowe W. C. Ginsberg J. (1996). Effects of the diacylglycerol kinase inhibitor, R59022, on TSH-stimulated iodide organification in porcine thyroid cells. Pharmacology 53, 376–383 9032802 10.1159/000139453

[B42] Mishkind M. Vermeer J. E. Darwish E. Munnik T. (2009). Heat stress activates phospholipase D and triggers PIP accumulation at the plasma membrane and nucleus. Plant J. 60, 10–21 10.1111/j.1365-313X.2009.03933.x 19500308

[B43] Mizoguchi M. Umezawa T. Nakashima K. Kidokoro S. Takasaki H. Fujita Y. (2010). Two closely related subclass II SnRK2 protein kinases cooperatively regulate drought-inducible gene expression. Plant Cell Physiol. 51, 842–847 10.1093/pcp/pcq041 20375108

[B44] Mizoi J. Ohori T. Moriwaki T. Kidokoro S. Todaka D. Maruyama K. (2013). GmDREB2A;2, a canonical DEHYDRATION-RESPONSIVE ELEMENT-BINDING PROTEIN 2-type transcription factor in soybean, is posttranslationally regulated and mediates dehydration-responsive element-dependent gene expression. Plant Physiol. 161, 346–361 10.1104/pp.112.204875 23151346 PMC3532265

[B45] Mogami H. Mills C. L. Gallacher D. V. (1997). Phospholipase C inhibitor, U73122, releases intracellular Ca^2+^, potentiates Ins(1 4, 5)P_3_-mediated Ca^2+^ release and directly activates ion channels in mouse pancreatic acinar cells. Biochem. J. 324, 645–651 9182729 10.1042/bj3240645 PMC1218477

[B46] Morse M. Rafudeen M. S. Farrant J. M. (2011). An overview of the current understanding of desiccation tolerance in the vegetative tissues of higher plants. Adv. Bot. Res. 57, 319–347 10.1016/B978-0-12-387692-387698.00009-6

[B47] Monteiro D. Liu Q. Lisboa S. Scherer G. E. F. Quader H. Malhó R. (2005). Phosphoinositides and phosphatidic acid regulate pollen tube growth and reorientation through modulation of [Ca2+]c and membrane secretion. J. Exp. Bot. 56, 1665–1674 10.1093/jxb/eri163 15837704

[B48] Mundree S. G. Baker B. Mowla S. Peters S. Marais S. Willigen C. V. (2002). Minireview - Physiological and molecular insights into drought tolerance. Afr. J. Biotechnol. 1, 28–38

[B49] Nakashima K. Ito Y. Yamaguchi-Shinozaki K. (2009). Transcriptional regulatory networks in response to abiotic stresses in Arabidopsis and Grasses. Plant Physiol. 149, 88–95 10.1104/pp.108.129791 19126699 PMC2613698

[B50] Nakashima K. Shinwari Z. K. Sakuma Y. Seki M. Miura S. Shinozaki K. (2000). Organization and expression of two Arabidopsis DREB2 genes encoding DRE-binding proteins involved in dehydration- and high-salinity-responsive gene expression. Plant Mol. Biol. 42, 657–665 10.1023/A:1006321900483 10809011

[B51] Parre E. Ghars M. A. Leprince A.-S. Thiery L. Lefebvre D. Bordenave M. (2007). Calcium signaling via phospholipase C is essential for proline accumulation upon ionic but not nonionic hyperosmotic stresses in Arabidopsis. Plant Physiol. 144, 503–512 10.1104/pp.106.095281 17369432 PMC1913778

[B52] Perera I. K. Hilmann I. Chang S. C. Boss W. F. Kaufman P. B. (2001). A role for inositol 1 4, 5-trisphosphate in gravitropic signaling and the retention of cold-perceived gravistimulation of oat shoot pulvini. Plant Physiol. 125, 1499–1507 10.1104/pp.125.3.1499 11244128 PMC65627

[B53] Perera I. Y. Hung C.-Y. Moore C. D. Stevenson-Paulik J. Boss W. F. (2008). Transgenic Arabidopsis plants expressing the type 1 Inositol 5-phosphatase exhibit increased drought tolerance and altered abscisic acid signaling. Plant Cell 20, 2876–2893 10.1105/tpc.108.061374 18849493 PMC2590728

[B54] Perera I. Y. Love J. Heilmann I. Thompson W. F. Boss W. F. (2002). Up-Regulation of phosphoinositide metabolism in tobacco cells constitutively expressing the human type I inositol polyphosphate 5-phosphatase. Plant Physiol. 129, 1795–1806 10.1104/pp.003426 12177493 PMC166768

[B55] Pokotylo I. Kolesnikov Y. Kravets V. Zachowski A. Ruelland E. (2014). Plant phosphoinositide-dependent phospholipases C: variations around a canonical theme. Biochimie. [Epub ahead of print]. 10.1016/j.biochi.2013.07.004 23856562

[B56] Potier M. Chantome A. Joulin V. Girault A. Roger S. Besson P. (2011). The SK3/K(Ca)2.3 potassium channel is a new cellular target for edelfosine. Br. J. Pharmacol. 162, 464–479 10.1111/j.1476-5381.2010.01044.x 20955368 PMC3031066

[B57] Powis G. Seewald M. J. Gratas C. Melder D. Riebow J. Modest E. J. (1992). Selective inhibition of phosphatidylinositol phospholipase C by cytotoxic ether lipid analogues. Cancer Res. 52, 2835–2840 1316230

[B58] Provart N. Zhu T. (2003). A browser-based functional classification SuperViewer for Arabidopsis genomics. Curr. Comput. Mol. Biol. 271–272

[B59] Qin C. Wang X. (2002). The Arabidopsis phospholipase D family. Characterization of a calcium-independent and phosphatidylcholine-selective PLD zeta 1 with distinct regulatory domains. Plant Physiol. 128, 1057–1068 10.1104/pp.010928 11891260 PMC152217

[B60] Rainteau D. Humbert L. Delage E. Vergnolle C. Cantrel C. Maubert M.-A. (2012). Acyl chains of Phospholipase D transphosphatidylation products in Arabidopsis cells: a study using multiple reaction monitoring mass spectrometry. PLoS ONE 7:e41985 10.1371/journal.pone.0041985 22848682 PMC3405027

[B61] Ramos-Díaz A. Brito-Argáez L. Munnik T. Hernández-Sotomayor S. M. (2007). Aluminum inhibits phosphatidic acid formation by blocking the phospholipase C pathway. Planta 225, 393–340 10.1007/s00425-006-0348-3 16821040

[B62a] Rhee S. Y. Beavis W. Berardini T. Z. Chen G. Dixon D. Doyle A. (2003). The Arabidopsis Information Resource (TAIR): a model organism database providing a centralized, curated gateway to Arabidopsis biology, research materials and community. Nucleic Acids Res. 31, 224–228 10.1093/nar/gkg076 12519987 PMC165523

[B62] Romano M. R. Lograno M. D. (2013). Signaling cross-talk between cannabinoid and muscarinic systems actives Rho-kinase and increases the contractile responses of the bovine ciliary muscle. Eur. J. Pharmacol. 702, 174–179 10.1016/j.ejphar.2013.01.053 23396229

[B63] Ruelland E. Cantrel C. Gawer M. Kader J.-C. Zachowski A. (2002). Activation of phospholipases C and D is an early response to a cold exposure in Arabidopsis suspension cells. Plant Physiol. 130, 999–1007 10.1104/pp.006080 12376663 PMC166625

[B64] Ruelland E. Vaultier M. Zachowski A. Hurry V. (2009). Cold signalling and cold acclimation in plants. Adv. Bot. Res. 49, 35–150 10.1016/S0065-229600602-2

[B65] Sakuma Y. Liu Q. Dubouzet J. G. Abe H. Shinozaki K. Yamaguchi-Shinozaki K. (2002). DNA-binding specificity of the ERF/AP2 domain of Arabidopsis DREBs, transcription factors involved in dehydration- and cold-inducible gene expression. Biochem. Biophys. Res. Commun. 290, 998–1009 10.1006/bbrc.2001.6299 11798174

[B66] Sakuma Y. Maruyama K. Qin F. Osakabe Y. Shinozaki K. Yamaguchi-Shinozaki K. (2006). Dual function of an Arabidopsis transcription factor DREB2A in water-stress-responsive and heat-stress-responsive gene expression. Proc. Natl. Acad. Sci. U.S.A. 103, 18822–18827 10.1073/pnas.0605639103 17030801 PMC1693746

[B67] Salinas-Mondragon R. E. Kajla J. D. Perera I. Y. Brown C. S. Sederoff H. W. (2010). Role of inositol 1 4, 5-triphosphate signalling in gravitropic and phototropic gene expression. Plant Cell Environ. 33, 2041–2055 10.1111/j.1365-3040.2010.02204.x 20584147

[B68] Sarkaria J. N. Tibbetts R. S. Busby E. C. Kennedy A. P. Hill D. E. Abraham R. T. (1998). Inhibition of phosphoinositide 3-kinase related kinases by the radiosensitizing agent wortmannin. Cancer Res. 58, 4375–4382 9766667

[B69] Schramm F. Larkindale J. Kiehlmann E. Ganguli A. Englich G. Vierling E. (2008). A cascade of transcription factor DREB2A and heat stress transcription factor HsfA3 regulates the heat stress response of Arabidopsis. Plant J. 53, 264–274 10.1111/j.1365-313X.2007.03334.x 17999647

[B70] Shedge V. Arrieta-Montiel M. Christensen A. C. Mackenzie S. A. (2007). Plant mitochondrial recombination surveillance requires unusual RecA and MutS homologs. Plant Cell 19, 1251–1264 10.1105/tpc.106.048355 17468263 PMC1913765

[B71] Shinozaki K. Yamaguchi-Shinozaki K. (2000). Molecular responses to dehydration and low temperature: differences and cross-talk between two stress signaling pathways. Curr. Opin. Plant Biol. 3, 217–223 10837265

[B72] Staxen I. Pical C. Montgomery L. T. Gray J. E. Hetherington A. M. McAinsh M. R. (1999). Abscisic acid induces oscillations in guard-cell cytosolic free calcium that involve phosphoinositide-specific phospholipase C. Proc. Natl. Acad. Sci. U.S.A. 96, 1779–1784 10.1073/pnas.96.4.1779 9990101 PMC15593

[B73] Stenzel I. Ischebeck T. König S. Hołubowska A. Sporysz M. Hause B. (2008). The type B phosphatidylinositol-4-phosphate 5-kinase 3 is essential for root hair formation in *Arabidopsis thaliana*. Plant Cell 20, 124–141 10.1105/tpc.107.052852 18178770 PMC2254927

[B74] Stevenson-Paulik J. Phillippy B. (2010). Inositol polyphosphates and kinases, in Lipid Signaling in Plants Plant Cell Monographs, ed Munnik T. (Berlin; Heidelberg: Springer), 161–174

[B75] Strassheim D. Shafer S. H. Phelps S. H. Williams C. L. (2000). Small cell lung carcinoma exhibits greater phospholipase C-beta1 expression and edelfosine resistance compared with non-small cell lung carcinoma. Cancer Res. 60, 2730–2736 10825148

[B76] Takahashi S. Katagiri T. Hirayama T. Yamaguchi-Shinozaki K. Shinozaki K. (2001). Hyperosmotic stress induces a rapid and transient increase in inositol 1 4, 5-trisphosphate independent of abscisic acid in Arabidopsis cell culture. Plant Cell Physiol. 42, 214–222 10.1093/pcp/pce028 11230576

[B77] Thole J. M. Nielsen E. (2008). Phosphoinositides in plants: novel functions in membrane trafficking. Curr. Opin. Plant Biol. 11, 620–631 10.1016/j.pbi.2008.10.010 19028349

[B78] Thyagarajan B. Benn B. S. Christakos S. Rohacs T. (2009). Phospholipase C-mediated regulation of transient receptor potential vanilloid 6 channels: implications in active intestinal Ca2+ transport. Mol. Pharmacol. 75, 608–616 10.1124/mol.108.052449 19073818 PMC2684912

[B79] Toyoda K. Kawahara T. Ichinose Y. Yamada T. Shiraishi T. (2000). Potentiation of phytoalexin accumulation in elicitor-treated epicotyls of Pea (*Pisum sativum*) by a diacylglycerol kinase inhibitor. J. Phytopath. 148, 633–636 10.1111/j.1439-0434.2000.00568.x

[B80] van der Luit A. H. Budde M. Ruurs P. Verheij M. van Blitterswijk W. J. (2002). Alkyl-lysophospholipid accumulates in lipid rafts and induces apoptosis via raft-dependent endocytosis and inhibition of phosphatidylcholine synthesis. J. Biol. Chem. 277, 39541–39547 10.1074/jbc.M203176200 12183451

[B81] Van Leeuwen W. Okrész L. Bögre L. Munnik T. (2004). Learning the lipid language of plant signalling. Trends Plant Sci. 9, 378–384 10.1016/j.tplants.2004.06.008 15358268

[B82] Van Leeuwen W. Vermeer J. E. M. Gadella T. W. J. Jr Munnik T. (2007). Visualization of phosphatidylinositol 4, 5-bisphosphate in the plasma membrane of suspension-cultured tobacco BY-2 cells and whole Arabidopsis seedlings. Plant J. 52, 1014–1026 10.1111/j.1365-313X.2007.03292.x 17908156

[B83] Vaultier M.-N. Cantrel C. Guerbette F. Boutté Y. Vergnolle C. Ciçek D. (2008). The hydrophobic segment of *Arabidopsis thaliana* cluster I diacylglycerol kinases is sufficient to target the proteins to cell membranes. FEBS Lett. 582, 1743–1748 10.1016/j.febslet.2008.04.042 18466768

[B84] Vergnolle C. Vaultier M.-N. Taconnat L. Renou J.-P. Kader J.-C. Zachowski A. (2005). The cold-induced early activation of phospholipase C and D pathways determines the response of two distinct clusters of genes in Arabidopsis cell suspensions. Plant Physiol. 139, 1217–1233 10.1104/pp.105.068171 16258011 PMC1283760

[B85] Vogler W. R. Shoji M. Hayzer D. J. Xie Y. P. Renshaw M. (1996). The effect of edelfosine on CTP:cholinephosphate cytidylyltransferase activity in leukemic cell lines. Leuk. Res. 20, 947–951 10.1016/S0145-212600070-7 9009253

[B86] Vorwerk S. Schiff C. Santamaria M. Koh S. Nishimura M. Vogel J. (2007). EDR2 negatively regulates salicylic acid-based defenses and cell death during powdery mildew infections of *Arabidopsis thaliana*. BMC Plant Biol. 7:35 10.1186/1471-2229-7-35 17612410 PMC1955445

[B87] Wang X. Devaiah S. P. Zhang W. Welti R. (2006). Signaling functions of phosphatidic acid. Prog. Lipid Res. 45, 250–278 10.1016/j.plipres.2006.01.005 16574237

[B88] Wiese A. Wieder T. Mickeleit M. Reinöhl S. Geilen C. C. Seydel U. (2000). Structure-dependent effects of glucose-containing analogs of platelet activating factor (PAF) on membrane integrity. Biol. Chem. 381, 135–144 10.1515/BC.2000.019 10746745

[B89] Williams M. E. Foster R. Chua N. H. (1992). Sequences flanking the hexameric G-box core CACGTG affect the specificity of protein binding. Plant Cell 4, 485–496 1498606 10.1105/tpc.4.4.485 PMC160147

[B90] Wilsher N. E. Court W. J. Ruddle R. Newbatt Y. M. Aherne W. Sheldrake P. W. (2007). The phosphoinositide-specific phospholipase C inhibitor U73122 (1-(6-((17β-3-Methoxyestra-1 3, 5-trien-17-yl)amino)hexyl)-1H-pyrrole-2, 5-dione) spontaneously forms conjugates with common components of cell culture medium. Drug Metab. Dispos. 35, 1017–1022 10.1124/dmd.106.014498 17403917

[B91] Wong R. Fabian L. Forer A. Brill J. A. (2007). Phospholipase C and myosin light chain kinase inhibition define a common step in actin regulation during cytokinesis. BMC Cell Biol. 8:15 10.1186/1471-2121-8-15 17509155 PMC1888687

[B92] Wymann M. P. Bulgarelli-Leva G. Zvelebil M. J. Pirola L. Vanhaesebroeck B. Waterfield M. D. (1996). Wortmannin inactivates phosphoinositide 3-kinase by covalent modification of Lys-802, a residue involved in the phosphate transfer reaction. Mol. Cell Biol. 16, 1722–1733 8657148 10.1128/mcb.16.4.1722 PMC231159

[B93] Yamaguchi-Shinozaki K. Shinozaki K. (1994). A novel cis-acting element in an Arabidopsis gene is involved in responsiveness to drought, low-temperature, or high-salt stress. Plant Cell 6, 251–264 10.1105/tpc.6.2.251 8148648 PMC160431

[B94] Yang Y. H. Dudoit S. Luu P. Lin D. M. Peng V. Ngai J. (2002). Normalization for cDNA microarray data: a robust composite method addressing single and multiple slide systematic variation. Nucleic Acids Res. 30:e15 10.1093/nar/30.4.e15 11842121 PMC100354

[B95] Yilmaz A. Mejia-Guerra M. K. Kurz K. Liang X. Welch L. Grotewold E. (2010). AGRIS: the Arabidopsis gene regulatory information server, an update. Nucleic Acids Res. 39, D1118–D1122 10.1093/nar/gkq1120 21059685 PMC3013708

[B96] Yoshida T. Sakuma Y. Todaka D. Maruyama K. Qin F. Mizoi J. (2008). Functional analysis of an Arabidopsis heat-shock transcription factor HsfA3 in the transcriptional cascade downstream of the DREB2A stress-regulatory system. Biochem. Biophys. Res. Commun. 368, 515–521 10.1016/j.bbrc.2008.01.134 18261981

[B97] Zhang Y. Xu W. Li Z. Deng X. W. Wu W. Xue Y. (2008). F-Box Protein DOR functions as a novel inhibitory factor for abscisic acid-induced stomatal closure under drought stress in Arabidopsis. Plant Physiol. 148, 2121–2133 10.1104/pp.108.126912 18835996 PMC2593669

[B98] Zhao Y. Yan A. Feijó J. A. Furutani M. Takenawa T. Hwang I. (2010). Phosphoinositides regulate clathrin-dependent endocytosis at the tip of pollen tubes in Arabidopsis and tobacco. Plant Cell 22, 4031–4044 10.1105/tpc.110.076760 21189293 PMC3027160

[B99] Zheng S. Z. Liu Y. L. Li B. Shang Z. L. Zhou R. G. Sun D.-Y. (2012). Phosphoinositide-specific phospholipase C9 is involved in the thermotolerance of Arabidopsis. Plant J. 69, 689–700 10.1111/j.1365-313X.2011.04823.x 22007900

[B100] Zhong L. R. Artinian L. Rehder V. (2013). Dopamine suppresses neuronal activity of Helisoma B5 neurons via a D2-like receptor, activating PLC and K channels. Neuroscience 228, 109–119 10.1016/j.neuroscience.2012.10.005 23069757

