# Supplementary material for: The Arabidopsis DREB2 genetic pathway is constitutively repressed by basal phosphoinositide-dependent phospholipase C coupled to diacylglycerol kinase
Source: Front Plant Sci. 2013 Aug 8;4:307. doi: 10.3389/fpls.2013.00307 (PMC3737466; doi:10.3389/fpls.2013.00307)
Supplement: Supplemental Table S2 — Contingency tables. (A) Contingency table of the expression of genes in response to U73122 and W30. (B) Contingency table of the expression of genes in response to edelfosine and R59022. (C) contingency table of the expression of genes in response to edelfosine and R59022 and W30. [file DataSheet2.DOCX]

**Supplemental Table S2A. Contingency table of the expression of genes in response to W30 or U73122 treatments.** For each category, the number of observed genes can be compared to that of theoretical genes, indicated between brackets, considering the expression of genes to the 2 molecules, separately, are independent. The ratio “number of observed genes” *vs.* “theoretical number” is in bold.

| Observed  (Theoretical)  **ratio** | U73122>U73343 | U73122<>U73343 | U73122<U73343 | total |
| --- | --- | --- | --- | --- |
| W30> W1 | 504  (91.79)  **5.49** | 677  (1007.01)  **0.67** | 20  (102.19)  **0.20** | 1201 |
| W30<> W1 | 1118  (1436.32)  **0.78** | 16409  (15757.55)  **1.04** | 1266  (125.68)  **0.79** | 18793 |
| W30< W1 | 19  (112.8)  **0.17** | 917  (1238.43)  **0.74** | 541  (3.64)  **4.30** | 1477 |
| total | 1641 | 18003 | 1827 | 21471 |

**Supplemental Table S2B. Contingency table of the expression of genes in response to edelfosine or R59022 treatments.** For each category, the number of observed genes can be compared to that of theoretical genes, indicated between brackets, considering the expression of genes to the 2 molecules, separately, are independent. The ratio “number of observed genes” *vs.* “theoretical number” is in bold.

| Observed  (Theoretical)  **ratio** | Edelfosine> control | Edelfosine<>control | Edelfosine< control | total |
| --- | --- | --- | --- | --- |
| R59022> control | 67  (2.92)  **23** | 115  (175.44)  **0.66** | 0  (3.64)  **0** | 182 |
| R59022<> control | 341  (407.34)  **0.84** | 24571  (24457.45)  **1** | 460  (507.20)  **0.91** | 25372 |
| R59022< control | 4  (1.73)  **2.31** | 51  (104.11)  **0.49** | 53  (2.16)  **24** | 108 |
| total | 412 | 24737 | 513 | 25662 |

**Table S2C. Contingency table of the expression of genes in response to edelfosine, R59002 or wortmannin treatments.** For each category, the number of observed genes can be compared to that of theoretical genes, indicated between brackets, considering the expression of genes to the 3 molecules, separately, are independent. The ratio “number of observed genes” *vs.* “theoretical number” is in bold.

| Observed  (Theoretical)  **ratio** | | R59022> control | R59022<> control | R59022< control | total |
| --- | --- | --- | --- | --- | --- |
| Edelfosine> control | W30 > W1 | 53  (1.7)  **31** | 175  (226.99)  **0.77** | 1  (1.01)  **0.99** | 229 |
|  | W30 <> W1 | 14  (1.29)  **10.85** | 156  (170.95)  **0.91** | 3  (0.76  **3.95** | 173 |
|  | W30 < W1 | 0  (0.07)  **0** | 10  (9.88)  **1.01** | 0  (0.04)  **0** | 10 |
| Edelfosine<> control | W30 > W1 | 74  (7.87)  **9.40** | 980  (1046.45)  **0.94** | 5  (4.67)  **1.07** | 1059 |
|  | W30 <> W1 | 41  (157.99)  **0.26** | 21178  (20999.25)  **1.01** | 32  (93.75)  **0.34** | 21251 |
|  | W30 < W1 | 0  (9.27)  **0** | 1233  (1232.23)  **1** | 14  (5.50)  **2.55** | 1247 |
| Edelfosine< control | W30 > W1 | 0  (0.09)  **0** | 11  (11.86)  **0.93** | 1  (0.05)  **20** | 12 |
|  | W30 <> W1 | 0  (0.99)  **0** | 128  (131.42)  **0.97** | 5  (0.59)  **8.48** | 133 |
|  | W30 < W1 | 0  (2.72)  **0** | 319  361.66)  **0.88** | 47  (1.61)  **29** | 366 |
|  | total | 182 | 24190 | 108 | 24480 |
