# Supplementary material for: The Arabidopsis DREB2 genetic pathway is constitutively repressed by basal phosphoinositide-dependent phospholipase C coupled to diacylglycerol kinase
Source: Front Plant Sci. 2013 Aug 8;4:307. doi: 10.3389/fpls.2013.00307 (PMC3737466; doi:10.3389/fpls.2013.00307)
Supplement: Supplemental Figure S1 — Venn diagrams. (A) Venn diagram representation of the overlaps between genes regulated by U73122 or edelfosine. (B) Venn diagram representation of the overlaps between genes regulated by W30 or edelfosine (Edel). (C) Venn diagram representation of the overlaps between genes regulated by R59022 or edelfosine (Edel). (D) Venn diagram representation of the overlaps between genes induced by W30, or R59022 or edelfosine (Edel). (E) Venn diagram representation of the overlaps between genes repressed by W30, or R59022 or edelfosine (Edel). (F) Venn diagram representation of the overlaps between genes regulated by R59022 or n-ButOH (nBut). (G) Venn diagram representation of the overlaps between genes regulated by n-ButOH (nBut) and edelfosine (Edel). + indicates genes induced by the molecule, − indicates genes repressed by the molecule. [file DataSheet4.PDF]

Supplemental figure S1. Venn diagrams. S1A, Venn diagram representation of the overlaps between genes regulated by U73122 or edelfosine. S1B, Venn diagram representation of the overlaps between genes regulated by W30 or edelfosine (Edel). S1C, Venn diagram representation of the overlaps between genes regulated by R59022 or edelfosine (Edel). S1D, Venn diagram representation of the overlaps between genes induced by W30, or R59022 or edelfosine (Edel). S1E, Venn diagram representation of the overlaps between genes repressed by W30, or R59022 or edelfosine (Edel). S1F. Venn diagram representation of the overlaps between genes regulated by R59022 or *n*-ButOH (nBut). S1G, Venn diagram representation of the overlaps between genes regulated by *n*-ButOH (nBut) and edelfosine (Edel). + indicates genes induced by the molecule, - indicates genes repressed by the molecule.

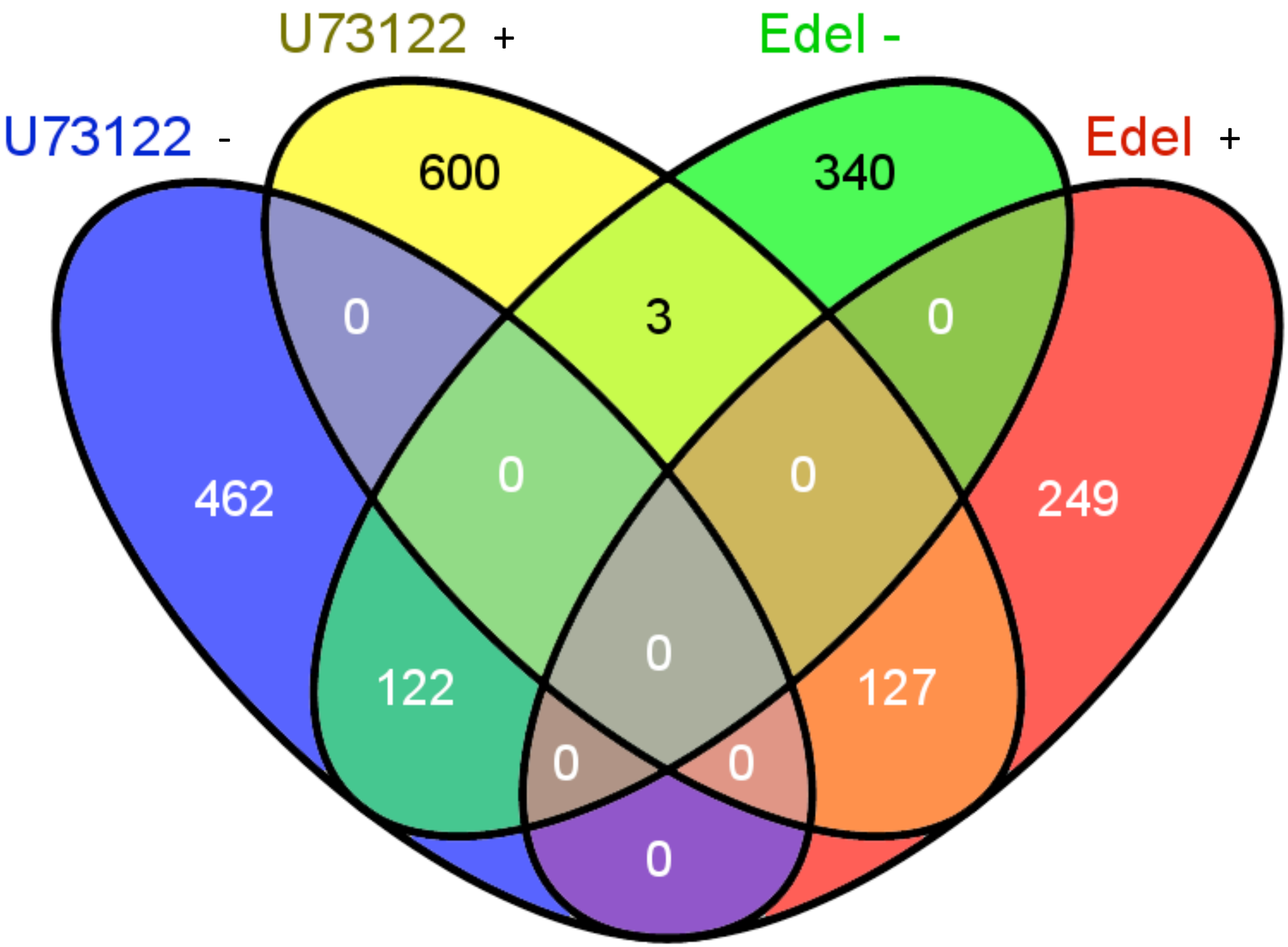

Supplemental figure S1A. Venn diagram representation of the overlaps between genes regulated by U73122 or edelfosine (Edel). + indicates genes induced by the molecule, - indicates genes repressed by the molecule.

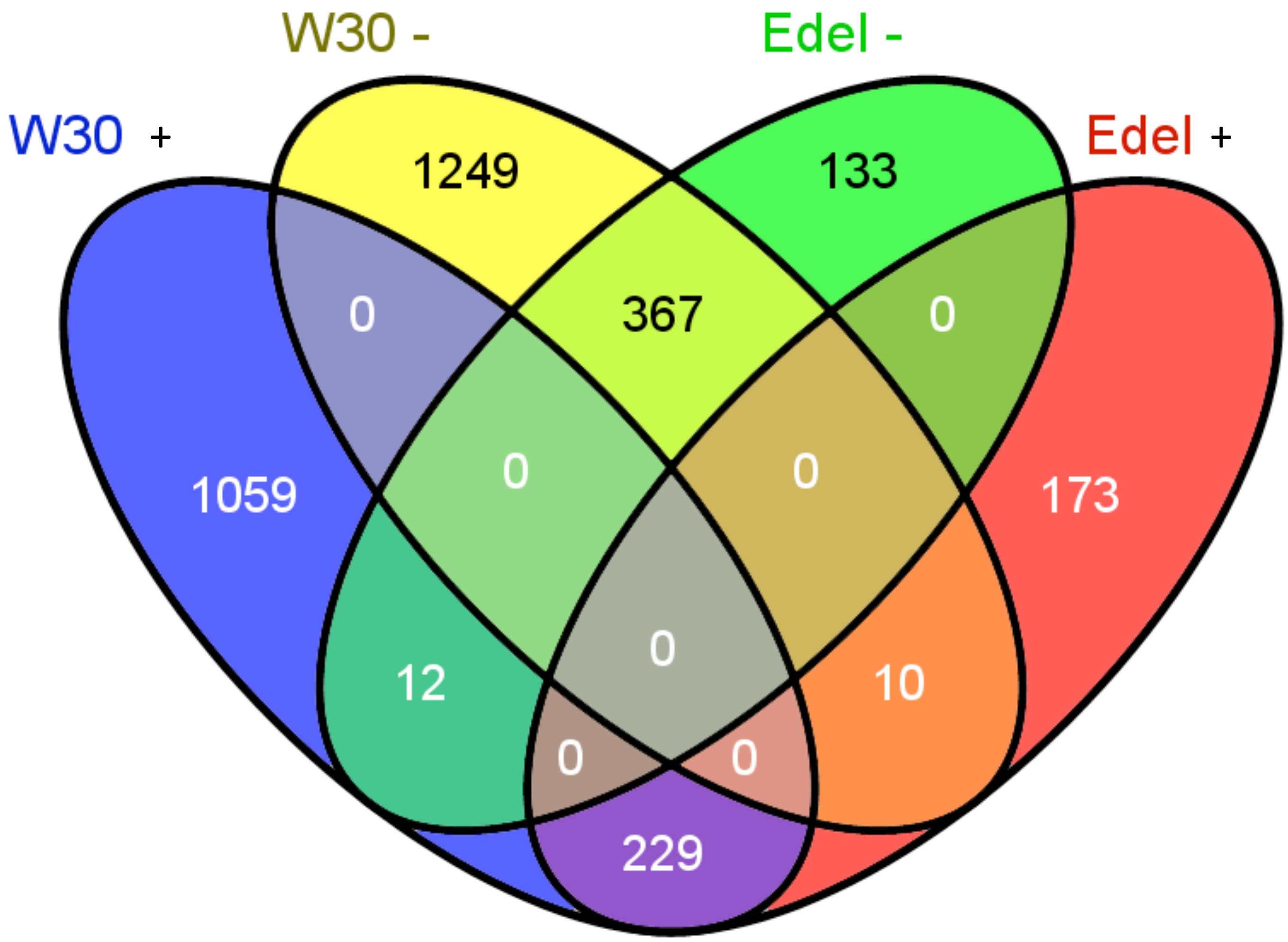

Supplemental figure S1B. Venn diagram representation of the overlaps between genes regulated by W30 and edelfosine (Edel). + indicates genes induced by the molecule, - indicates genes repressed by the molecule.

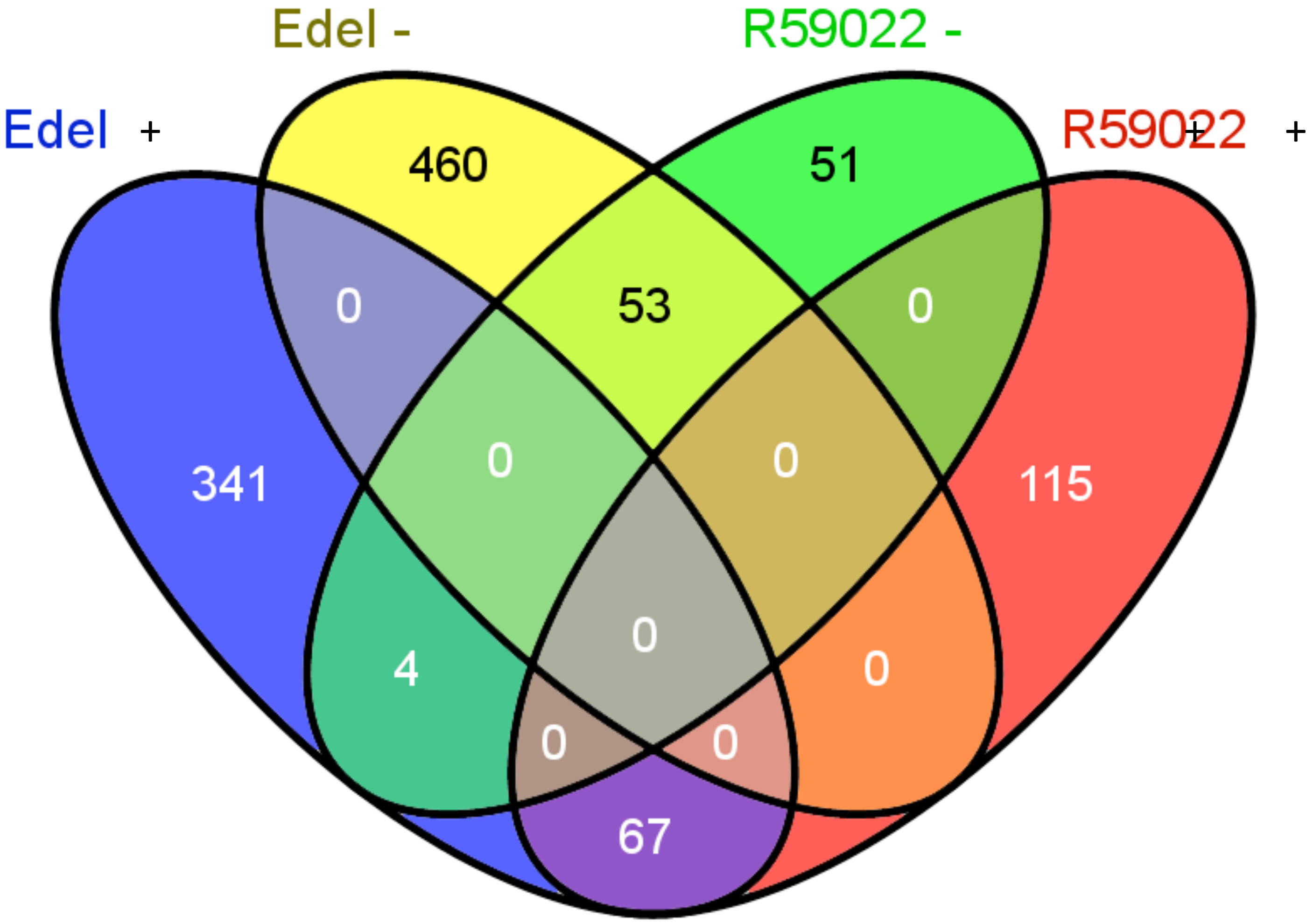

Supplemental figure S1C. Venn diagram representation of the overlaps between genes regulation by R59022 and edelfosine (Edel). + indicates genes induced by the molecule, - indicates genes repressed by the molecule.

Supplemental figure S1D. Venn diagram representation of the overlaps between genes induced by W30, or R59022 or edelfosine (Edel).

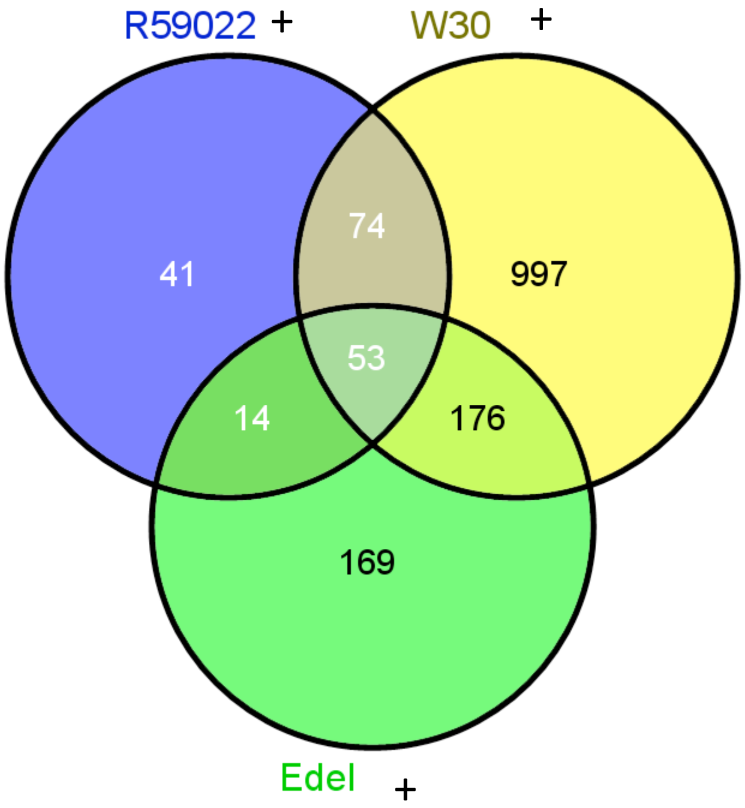

Supplemental figure S1E. Venn diagram representation of the overlaps between genes repressed by W30, or R59022 or edelfosine (Edel).

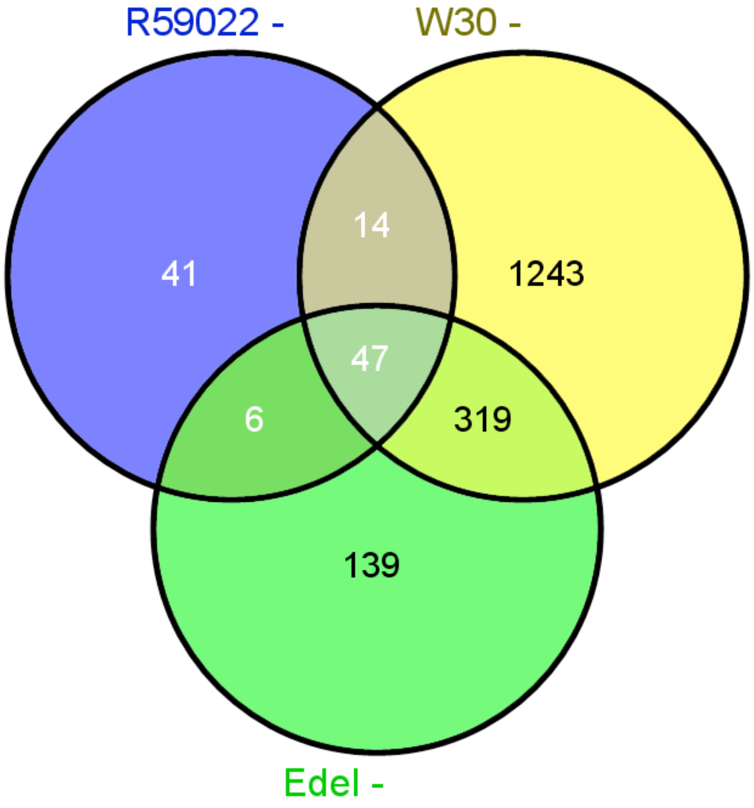

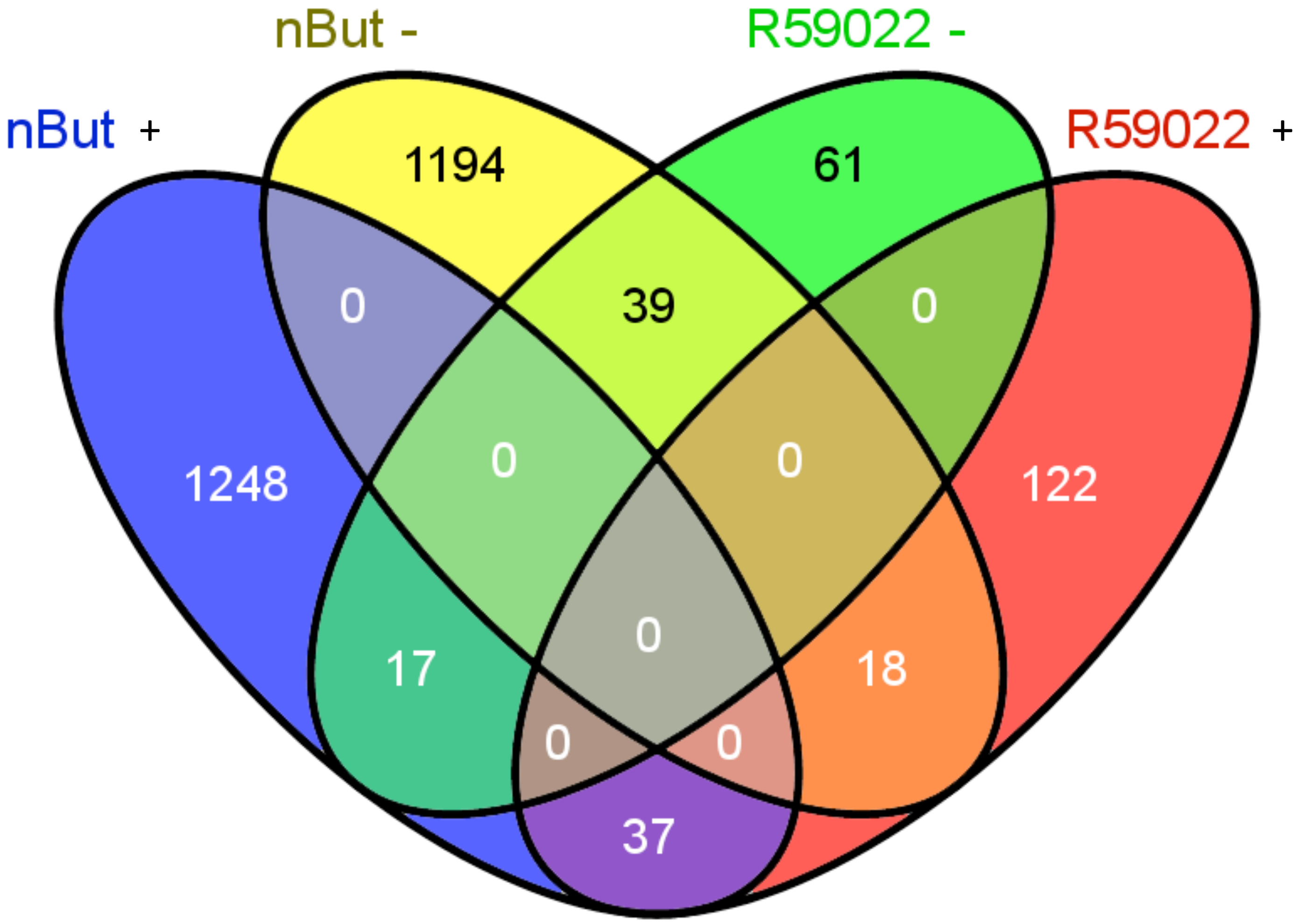

Supplemental figure S1F. Venn diagram representation of the overlaps between genes regulated by R59022 and *n*-ButOH (nBut). + indicates genes induced by the molecule, - indicates genes repressed by the molecule.

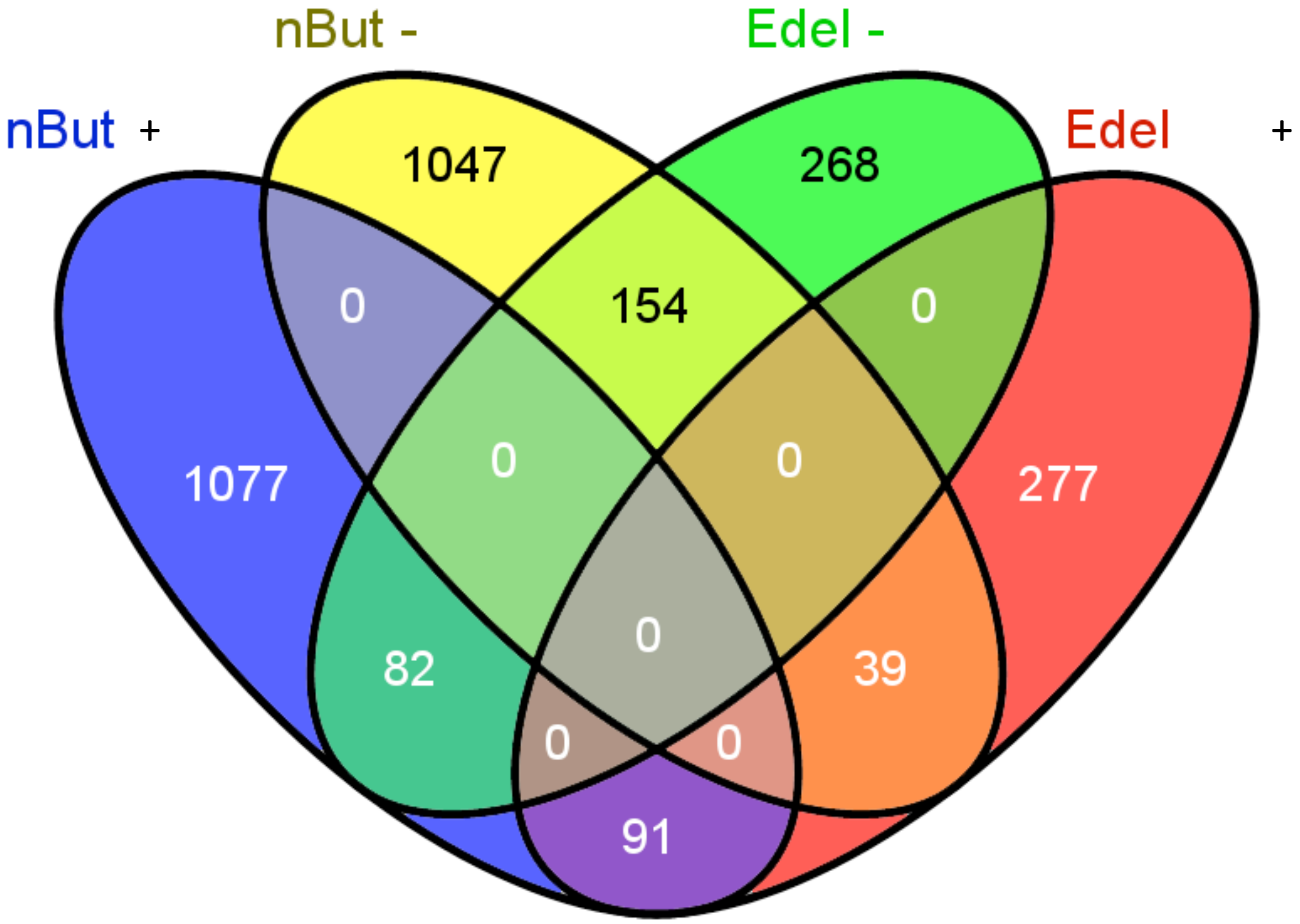

Supplemental figure S1G. Venn diagram representation of the overlaps between genes regulated by *n*-ButOH (nBut) and edelfosine (Edel). + indicates genes induced by the molecule, - indicates genes repressed by the molecule.
