# Supplementary material for: The Arabidopsis DREB2 genetic pathway is constitutively repressed by basal phosphoinositide-dependent phospholipase C coupled to diacylglycerol kinase
Source: Front Plant Sci. 2013 Aug 8;4:307. doi: 10.3389/fpls.2013.00307 (PMC3737466; doi:10.3389/fpls.2013.00307)
Supplement: Supplemental Figure S2 — Working models of PI-PLC actions. (A) In resting cells, basal PI-PLCs produce InsP3 that can be transformed into other phosphorylated inositols, and DAG that can be phosphorylated, into PA. Phosphorylated inositols and PA can activate transducing pathways that lead to DREB2 gene basal down-regulation. (B) Simplified version of (A), to be used for comparison with other panels. (C) When the basal PI-PLCs are inhibited, this leads to a decrease in products, concomitantly with an increase in the substrates, the phosphorylated PI (named phosphoinositides). (D) The first committing enzymes phosphorylating PI into the phosphoinositides substrates of PI-PLCs are the type III-PI4K (Delage et al., 2012 and this article). Their inhibition by 30 μ M wortmannin leads to a decrease in phosphoinositides but also in PI-PLC products. (E) PI-PLC activation would lead to a decrease of the phosphoinositide pools, concomitantly with an increase of PI-PLC products. (F) In some stress situations, a PI-PLC has been reported to be activated, but associated with an increase of phosphoinositides, which can happen if the phosphoinositide-producing pathway is activated at a higher rate than the PI-PLC activation. [file DataSheet5.PDF]

Supplemental figure S2. Working models of PI-PLC actions. A, In resting cells, basal PI-PLCs produce InsP3 that can be transformed into other phosphorylated inositols, and DAG that can be phosphorylated, into PA. Phosphorylated inositols and PA can activate transducing pathways that lead to DREB2 gene basal down-regulation. B, simplified version of A, to be used for comparison with other panels. C, When the basal PI-PLCs are inhibited, this leads to a decrease in products, concomitantly with an increase in the substrates, the phosphorylated PI (named phosphoinositides). D, The first committing enzymes phosphorylating PI into the phosphoinositides substrates of PI-PLCs are the type III-PI4K (Delage et al., 2012 and this article). Their inhibition by 30 µM wortmannin leads to a decrease in phosphoinositides but also in PI-PLC products. E, PI-PLC activation would lead to a decrease of the phosphoinositide pools, concomitantly with an increase of PI-PLC products. F, in some stress situations, a PI-PLC has been reported to be activated, but associated with an increase of phosphoinositides, which can happen if the phosphoinositide-producing pathway is activated at a higher rate than the PI-PLC activation.

A. Resting cells

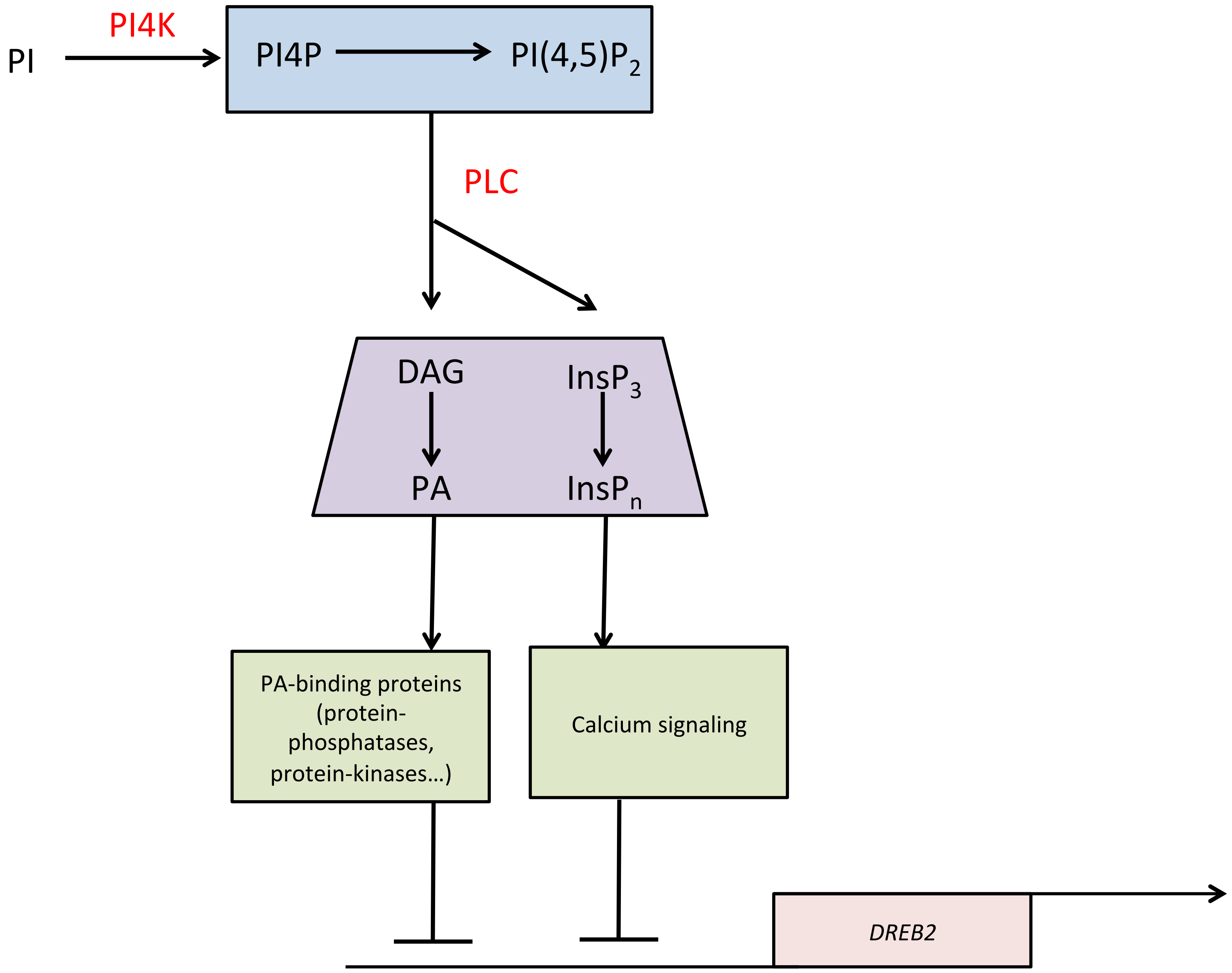

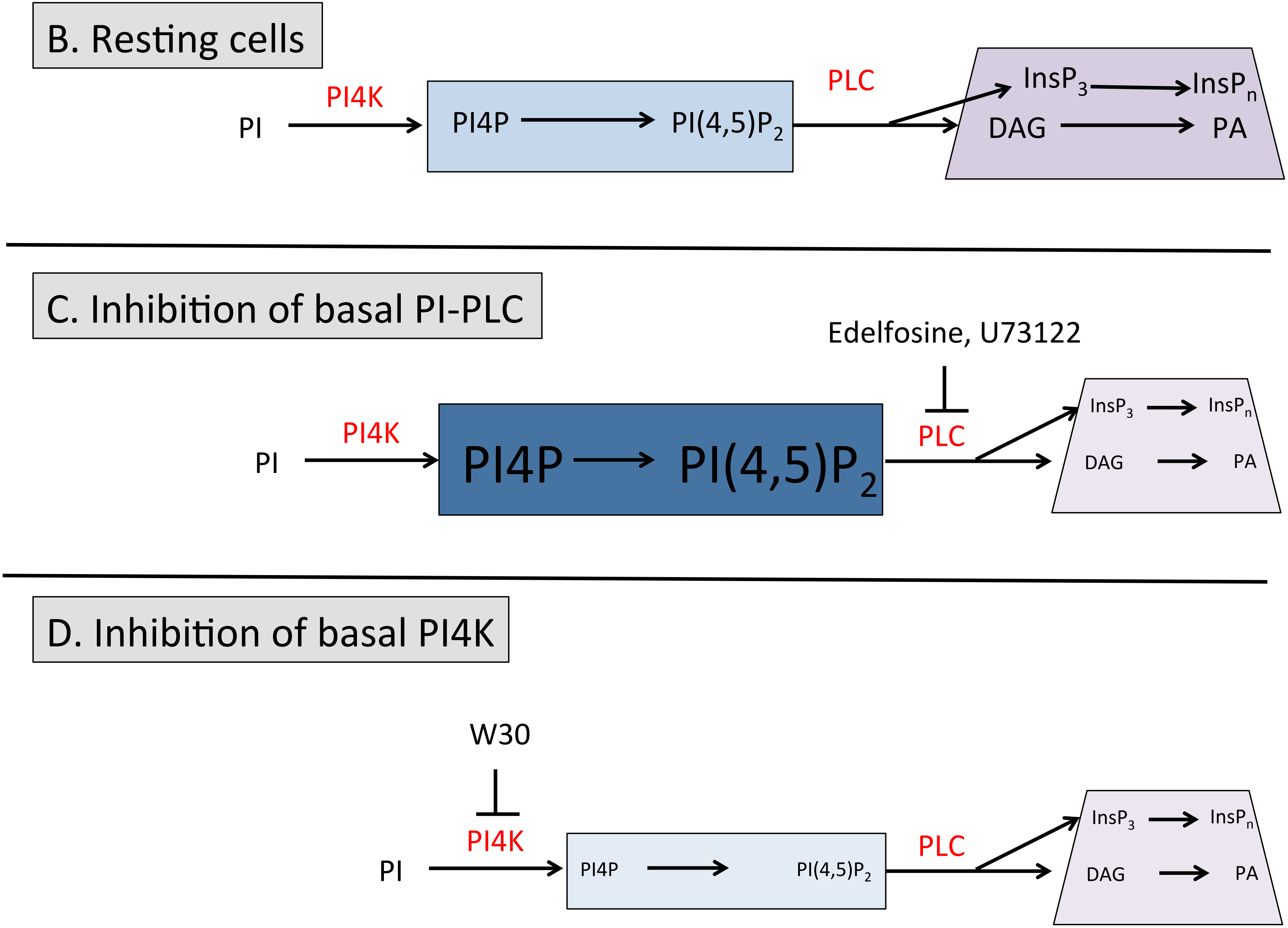

B. Resting cells
PI
PI4K
PI4P
PI(4,5)P2
PLC
InsP3
InsPn
DAG
PA
C. Inhibition of basal PI-PLC
Edelfosine, U73122
PI
PI4K
PI4P
PI(4,5)P2
PLC
InsP3
InsPn
DAG
PA
D. Inhibition of basal PI4K
W30
PI
PI4K
PI4P
PI(4,5)P2
PLC
InsP3
InsPn
DAG
PA

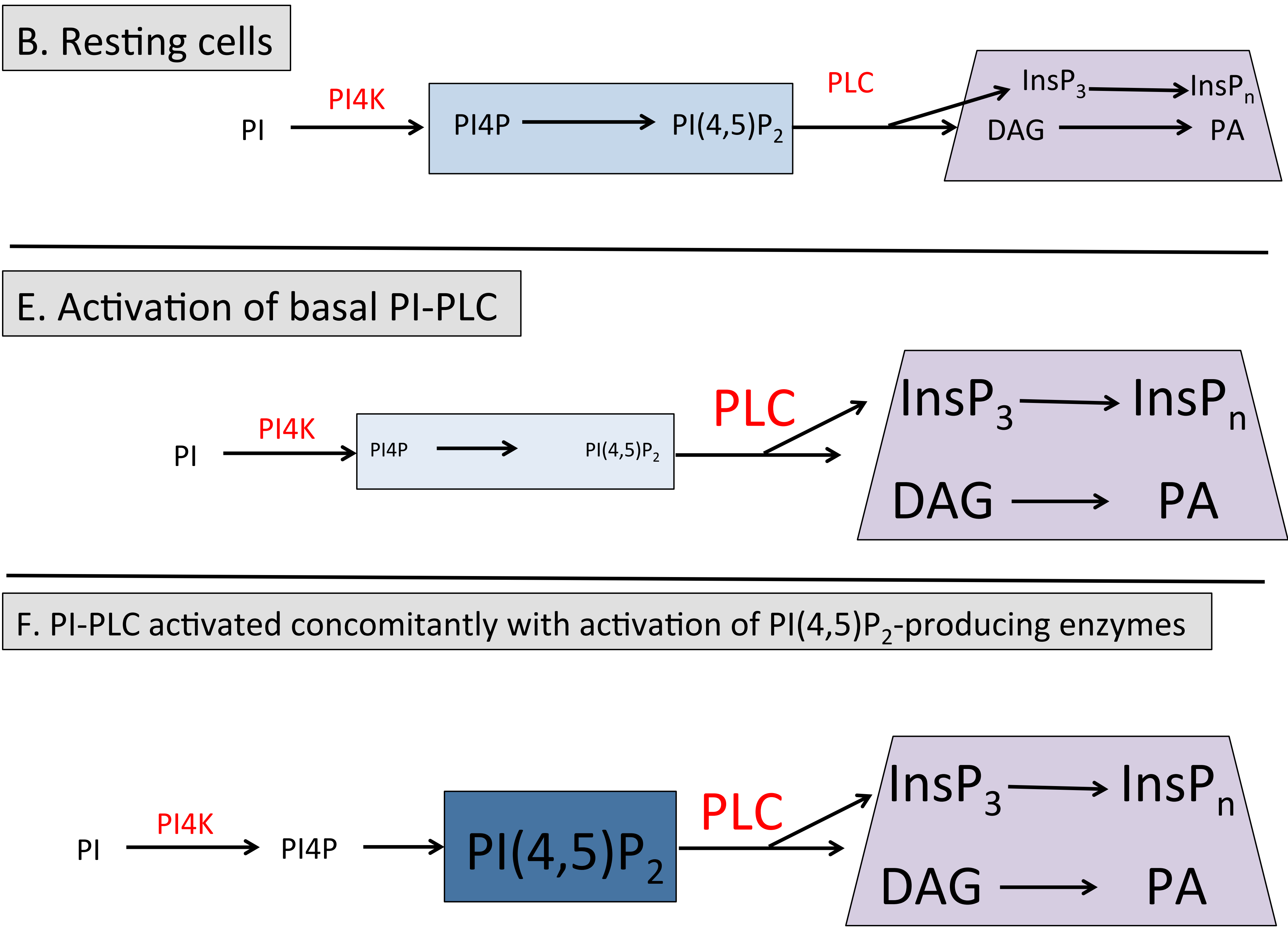
B. Resting cells
PI
PI4K
PI4P
PI(4,5)P2
PLC
InsP3
InsPn
DAG
PA
E. Activation of basal PI-PLC
PI
PI4K
PI4P
PI(4,5)P2
PLC
InsP3
InsPn
DAG
PA
F. PI-PLC activated concomitantly with activation of PI(4,5)P2-producing enzymes
PI
PI4K
PI4P
PI(4,5)P2
PLC
InsP3
InsPn
DAG
PA
