# Supplementary material for: The Arabidopsis DREB2 genetic pathway is constitutively repressed by basal phosphoinositide-dependent phospholipase C coupled to diacylglycerol kinase
Source: Front Plant Sci. 2013 Aug 8;4:307. doi: 10.3389/fpls.2013.00307 (PMC3737466; doi:10.3389/fpls.2013.00307)

Supplemental Figure S3. Classification of edelfosine- and W30- induced or repressed genes according to their biological processes. For each biological process, the ratio to whole Arabidopsis set (genome) is shown. In grey bars are the categories not statistically different from the whole Arabidopsis set. Bars in bright colours are for the over-represented categories and that in shaded colours the under-represented ones.

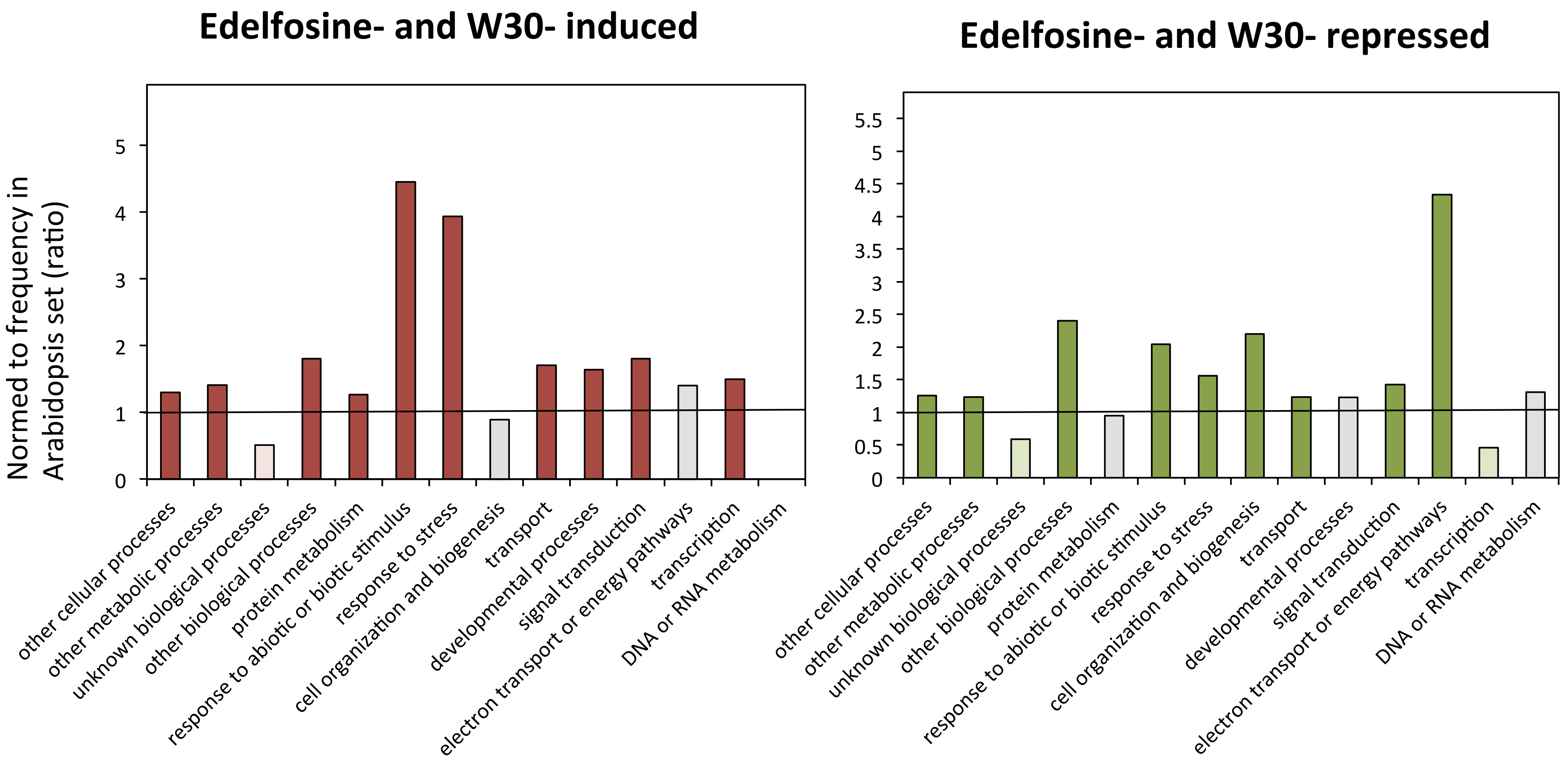

Supplement: Supplemental Figure S3 — Classification of edelfosine- and W30-induced or repressed genes according to their biological processes. For each biological process, the ratio to whole Arabidopsis set (genome) is shown. In gray bars are the categories not statistically different from the whole Arabidopsis set. Bars in bright colors are for the over-represented categories and that in shaded colors the under-represented ones. [file DataSheet6.PDF]
