# Supplementary material for: The Arabidopsis DREB2 genetic pathway is constitutively repressed by basal phosphoinositide-dependent phospholipase C coupled to diacylglycerol kinase
Source: Front Plant Sci. 2013 Aug 8;4:307. doi: 10.3389/fpls.2013.00307 (PMC3737466; doi:10.3389/fpls.2013.00307)
Supplement: Supplemental Figure S4 — Effects of R59022 on the expression levels of DREB2 genes and target genes in seedlings. Twelve-day old plants grown in liquid medium under continuous light were incubated with inhibitors and harvested at desired times. Transcript levels were estimated by reverse transcriptase-PCR, using an appropriate number of cycles. [file DataSheet7.PDF]

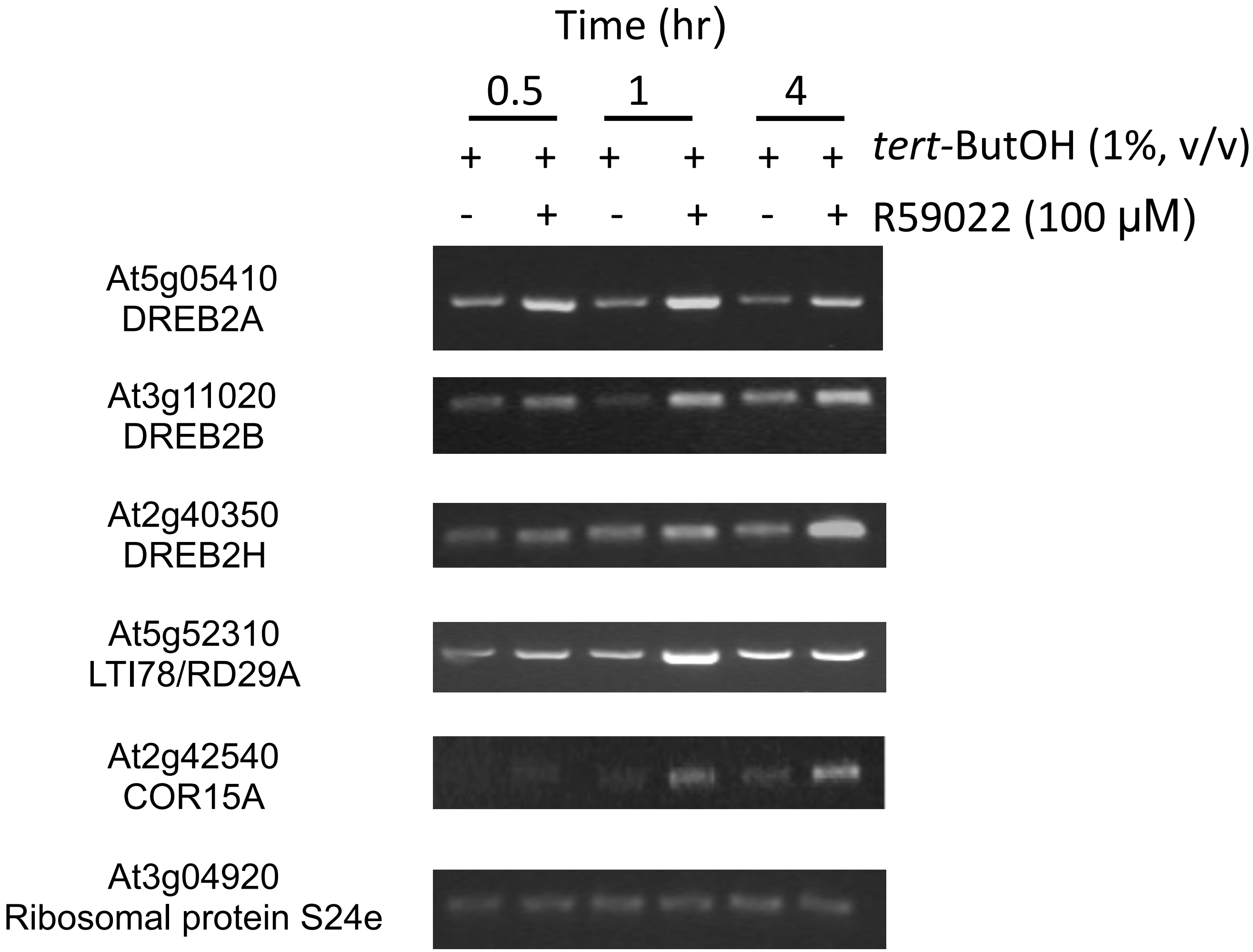

Supplemental Figure S4. Effects of R59022 on the expression levels of *DREB2* genes and target genes in seedlings. Twelve-day old plants grown in liquid medium under continuous light were incubated with inhibitors and harvested at desired times. Transcript levels were estimated by reverse transcriptase-PCR, using an appropriate number of cycles.
